# Regulation of DNA Replication within the Immunoglobulin Heavy-Chain Locus During B Cell Commitment

**DOI:** 10.1371/journal.pbio.1001360

**Published:** 2012-07-10

**Authors:** Agnieszka Demczuk, Michel G. Gauthier, Ingrid Veras, Settapong Kosiyatrakul, Carl L. Schildkraut, Meinrad Busslinger, John Bechhoefer, Paolo Norio

**Affiliations:** 1 Department of Oncology, Montefiore Medical Center, Moses Division, Bronx, New York, United States of America; 2 Department of Physics, Simon Fraser University, Burnaby, Canada; 3 Department of Cell Biology, Albert Einstein College of Medicine, Bronx, New York, United States of America; 4 Research Institute of Molecular Pathology, Vienna Biocenter, Vienna, Austria; 5 Department of Medicine, Albert Einstein College of Medicine, Bronx, New York, United States of America; National Cancer Institute, United States of America

## Abstract

The temporal order of replication of mammalian chromosomes appears to be linked to their functional organization, but the process that establishes and modifies this order during cell differentiation remains largely unknown. Here, we studied how the replication of the *Igh* locus initiates, progresses, and terminates in bone marrow pro-B cells undergoing B cell commitment. We show that many aspects of DNA replication can be quantitatively explained by a mechanism involving the stochastic firing of origins (across the S phase and the *Igh* locus) and extensive variations in their firing rate (along the locus). The firing rate of origins shows a high degree of coordination across *Igh* domains that span tens to hundreds of kilobases, a phenomenon not observed in simple eukaryotes. Differences in domain sizes and firing rates determine the temporal order of replication. During B cell commitment, the expression of the B-cell-specific factor Pax5 sharply alters the temporal order of replication by modifying the rate of origin firing within various *Igh* domains (particularly those containing Pax5 binding sites). We propose that, within the *Igh* C_H_-3′RR domain, Pax5 is responsible for both establishing and maintaining high rates of origin firing, mostly by controlling events downstream of the assembly of pre-replication complexes.

## Author Summary

Each time a mammalian cell duplicates its genome in preparation for cell division it activates thousands of so called “DNA origins of replication.” The timely and complete duplication of the genome depends on careful orchestration of origin activation, which is modified when cells differentiate to perform a specific function. We currently lack a universally accepted model of origin regulation that can explain the replication dynamics in complex eukaryotes. Here, we studied the mouse immunoglobulin heavy-chain locus, one of the antibody-encoding portions of the genome, where origins change activity when antibody-producing B cells differentiate in the bone marrow. We show that multiple aspects of DNA replication initiation, progression, and termination can be explained mathematically by the interplay between randomly firing origins and two independent variables: the speed of progression of replication forks and the firing rate of origins along the locus. The rate of origin firing varies extensively along the locus during B cell differentiation and, thus, is a dominant factor in establishing the temporal order of replication. A differentiation factor called Pax5 can alter the temporal order of replication by modifying the rate of origin firing across various parts of the locus.

## Introduction

During the S phase, mammalian chromosomes replicate in a precise temporal order, with the timing of replication typically changing gradually across hundreds of kilobases. Cell differentiation induces regional changes in the order of replication which can affect 45%, or more, of the mouse genome [1]. Various studies have examined how the temporal order of replication is established and modified at specific gene loci, but provided discordant explanations about the role played by DNA origins of replication. For example, within a 340 kb portion of the *Igh* locus, changes in replication timing have been linked to modifications in the distribution of active origins and in their firing efficiency (see definitions in Table 1) [2]. In contrast, within the *beta-globin* locus, changes in replication timing can occur without significant changes in origin distribution, or firing efficiency, and have been ascribed to modifications in the timing of origin firing [3]–[5]. Does this mean that the temporal order of replication is determined by multiple mechanisms? Are origin distribution, firing efficiency, and the timing of origin firing regulated independently? Which aspect of origin activation is controlled by cell differentiation? These are some of the questions addressed in this study.

**Table 1:** Definitions for various terms used in the text.

**Origin of Replication**	Any genomic site at which DNA replication can begin. In some organisms, these sites can be identified from their DNA sequence; in others they cannot.
**Origin Density**	The average number of distinct potential origin of replication present within a genomic regions of specified size in a defined population of cells. This definition implies that replication complexes must form at each of these sites in at least a fraction of the cell population (but not necessarily at every potential origin in each individual cell of the population).
**Origin Firing**	Activation of bidirectional replication forks at a potential origin.
**Passive Replication**	Replication of a potential origin by a fork generated elsewhere.
**Origin Efficiency**	The parameter most frequently used to indicate the level of origin activity (e.g., the relative intensity of bubble arcs in neutral-neutral 2D-gel experiments). For a genomic region of defined size and location, it represents the ratio between the number of firing events and the total number of replication events (firing events + passive replication). In other words, it expresses the frequency at which origins fire within the region during each S phase. Since this value is affected by external events (causing the passive replication of the region), it is not an intrinsic property of origins.When a genomic region contains a single origin, efficiency is always a value between 0 and 1. For example, a value of 0.5 indicates that during each S phase a specific origin fires in 50% of the cells.In contrast, if the region contains multiple origins the value is always ≥0. In the article, we refer to this latter value with the acronym IAS (initiations per allele per S phase).
**Timing of Origin Firing**	A common interpretation of the replication dynamics in eukaryotic cells assumes that specific origins fire at specific times during the S phase.
**Stochastic Origin Firing**	The model used for the analysis of our experimental data is stochastic in that it assumes that potentially active origins are present at high density throughout the *Igh* locus and that firing can take place in any unreplicated portion of the locus at any time during the S phase. The rate at which origins fire along the locus can vary and is determined by the cellular context.
**Origin Firing Rate**	For an unreplicated portion of the genome of defined size and location, the firing rate indicates the number of origin firings per unit of time that occur as cells transit through the S phase. This value measures an intrinsic property of origins within specific portions of the genome, which persists from the time of the G1/S transition, through the S phase, until the region is fully replicated.For example, a value of 500×10^−6^ initiations/kb/min indicates that, within a specific 1 kb section of the genome, 500 origins will fire every minute in a population of 10^6^ unreplicated DNA molecules (alleles).

Answering these questions requires a quantitative understanding of the dynamics of origin firing. Based on measurements of average origin activity across entire genomes, various stochastic models of origin firing have been recently used to explain specific aspects of eukaryotic DNA replication, such as the duration of S phase [6]–[13]. If origin firing can occur stochastically anywhere along the genome and at any time during S phase, origin distribution and the timing of origin firing cannot be responsible for establishing the temporal order of replication [14]. Recent observations indicate that the profile of replication timing of the budding yeast genome can be explained by differences in the firing rate of individual origins and stochastic origin firing [15]. However, yeast differs from metazoans in many aspect of DNA replication (e.g., *S. cerevisiae* has well-defined origins of replication, lacks the developmental control of the temporal order of replication, shows no correlation between gene expression and the temporal order of replication, has a short S phase, etc.). In addition, previous studies have mostly relied on the measurement of individual parameters of DNA replication which can be modeled with limited detail to determine the dynamics of origin firing (e.g., the timing of replication) and can produce misleading results if applied to complex genomes [16]. Hence, testing this hypothesis rigorously in mammalian cells requires the measurement of multiple parameters of DNA replication.

**Figure 1:**
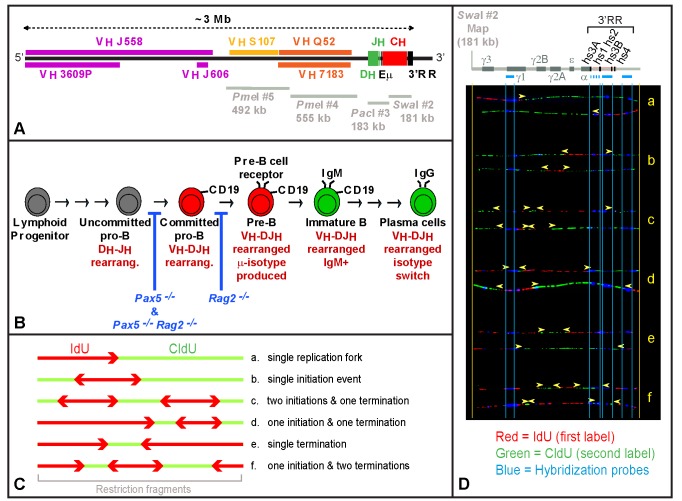
Experimental system and approach used in this study. (A) Map of the *Igh* locus (129/Sv) with approximate positions of the various gene families—constant (C_H_), joining (J_H_), diversity (D_H_), proximal-variable (proximal-V_H_; *V_H_7183*, *V_H_Q52*), middle-variable (middle-V_H_; *V_H_S107*), distal-variable (distal-V_H_; *V_H_J606*, *V_H_J558*, *V_H_3609P*)—and regulatory elements—intronic enhancer (Eμ), and 3′ regulatory region (3′RR). Gray bars indicate the positions of the four restriction fragments analyzed in this study. Note that while *PmeI* #5 and *PmeI* #4 span a continuous portion of the locus, a gap of ∼20 kb is present between the right end of *PmeI* #4 and the left end of *PacI* #3. A short overlap is also present between *PacI* #3 and *SwaI* #2. (B) Schematic representation of B cell development. Rag2 mediates the step of DNA cleavage during D_H_-J_H_ and V_H_-DJ_H_ recombination. When this gene is mutated, cells cannot assemble the pre-B cell receptor and are unable to develop beyond the committed pro-B stage of differentiation. In contrast, Pax5 regulates the expression of hundreds of genes involved in B cell commitment, and in its absence, cell differentiation stops at the uncommitted pro-B cell stage. (C) SMARD. *a–f* represent examples of staining patterns for six hypothetical double-labeled DNA molecules. Diverging and converging forks indicate the occurrence of initiation and termination events. (D) Images of 12 actual molecules representative of the categories depicted in panel C. Each image is aligned to the map of the corresponding genomic fragment using the position of “landmark” hybridization probes as a reference. Arrowheads mark the position of the IdU-CldU transitions.

Any large portion of a mammalian genome can be used to test the stochastic firing of origins provided enough information is available about DNA replication. For this reason, we used the assay called single molecule analysis of replicated DNA (SMARD) [17] to collect unbiased information about all aspects of DNA replication initiation, progression, and termination across a 1.4 megabase region encompassing the mouse immunoglobulin heavy-chain (*Igh*) locus (Figure 1A). The experimental data sets collected by SMARD included the temporal order of replication, the steady-state distribution of replication forks, the time required to replicate the region, the average speed of replication forks, the distribution of initiation and termination events, the percentage of replicating molecules containing initiation and termination events, and the average number of events per molecule.

Using a novel mathematical procedure [18], we established that the experimental data sets collected by SMARD are fully consistent with the stochastic firing of origins as defined in Table 1. We also show that many aspects of DNA replication (including the temporal order of replication) can be explained by variations in the rate of origin firing *I(x,t)* along the *Igh* locus (see definition in Table 1). According to the nomenclature proposed by others [19], this rate indicates the number of initiation events occurring per length of unreplicated DNA, over a given period of time, as mathematically defined in Materials and Methods.

Our results point to significant differences in the regulation of DNA replication between the mouse *Igh* locus and yeast chromosomes. In *S. cerevisiae*, each origin of replication appears to be characterized by a specific firing rate, which differs for different origins [15]. Within the *Igh* locus, initiation events lack focal points corresponding to individual origins. Instead, the locus comprises large domains (spanning tens or hundreds of kilobases) where the firing rate of multiple origins is virtually uniform and similarly regulated. Along the locus, changes in the firing rate occur abruptly at the border between different *Igh* domains, and the firing rate of each domain is not affected by deletions that span multiple origins. It is the combined effect of different domain sizes and firing rates that determines the temporal order of replication. This organization remains valid for cells at different stages of B cell development (e.g., bone marrow pro-B cells blocked at the uncommitted and committed stages of differentiation by homozygous mutations of the *Pax5* and *Rag2* genes; Figure 1B). We also show that the changes in DNA replication that occur during B cell commitment can be quantitatively explained by substantial changes in the firing rate of origins within specific domains of the *Igh* locus. Therefore, the rate of origin firing is the parameter that is being regulated across large sections of the locus during cell differentiation. The role of the developmental regulator Pax5 in this process and its mechanism of action are also examined.

## Results

### In Uncommitted Pro-B Cells the Replication of the *Igh* Locus Follows a Precise Temporal Order of Replication

Bone marrow pro-B cells isolated from *Pax5* ^−*/*−^ *Rag2* ^−*/*−^ mice retain the ability to proliferate but are blocked at the uncommitted stage of differentiation and maintain the *Igh* locus in germline configuration [20]. In order to perform SMARD, we sequentially labeled a population of exponentially growing cells with 5′-iodo-2′-deoxyuridine (IdU) and 5′-chloro-2′-deoxyuridine (CldU), for 3–4 h (see Table S1, column b). Under these conditions, each labeling period is long enough to allow the complete replication of large sections of the *Igh* locus, resulting in DNA molecules variably substituted with the halogenated nucleotide (see example in Figure S1) [2],[17],[21],[22]. We then digested the genomic DNA with restriction enzymes that cut infrequently within the locus (*PmeI*, or *PacI*, or *SwaI*) and isolated four of the resulting restriction fragments by pulsed-field gel electrophoresis (gray bars in Figure 1A). These fragments were stretched on microscope slides, hybridized with specific DNA probes, and analyzed by fluorescence microscopy to detect the incorporation of the nucleotide analogs along individual DNA molecules.

The molecules fully substituted with halogenated nucleotides provide a vast amount of information about the process of DNA replication [2],[17],[21],[22]. In *Pax5* ^−*/*−^ *Rag2* ^−*/*−^ cells, the analysis of ∼4,000 hybridization signals yielded 1,304 fully substituted DNA molecules, 764 of which met the standards required to perform precise measurements (Table S1, columns c–f). This population includes molecules that incorporated only one type of halogenated nucleotide (single-labeled molecules), as well as molecules that incorporated both of them (double-labeled DNA molecules). As explained in previous publications [17],[22], the ratio between single- and double-labeled molecules is proportional to the time required to replicate each restriction fragment (Table S1), which is linked to the average number of replication forks participating in the replication of each restriction fragment and to their average speed (Table S1). Thus, these values can be directly determined from the experimental data as described in the legend of Table S1. This allowed us to determine that, in uncommitted pro-B cells, the average speed of replication forks is similar within the four restriction fragments (between 2.25 and 3 kb/min). A similar value was also measured at the genomic level using a different assay (∼2.5 kb/min; IV and PN, unpublished observation). This suggests that replication forks move at comparable speeds throughout the *Igh* locus.

**Figure 2:**
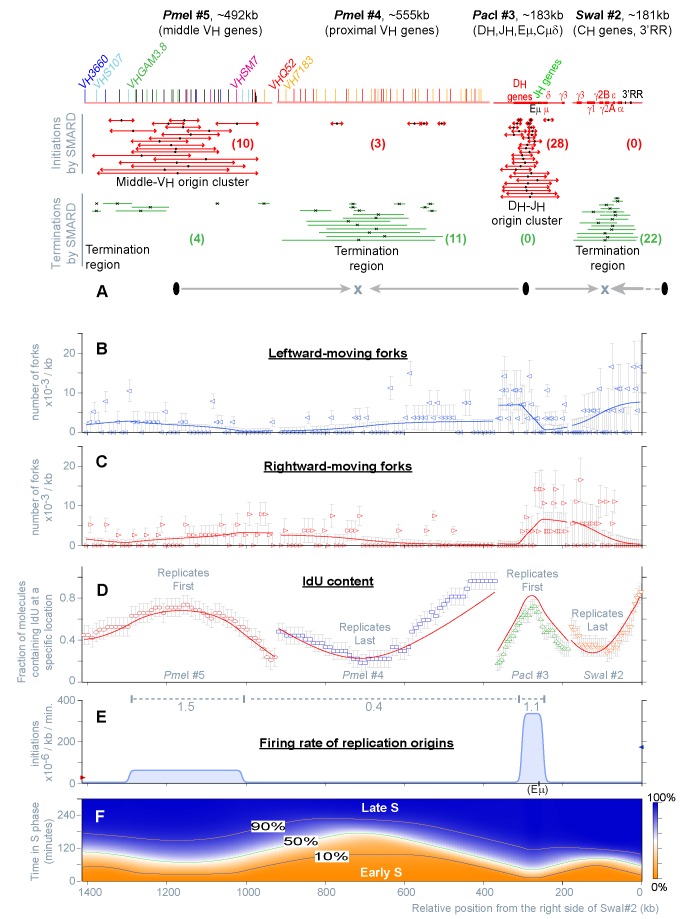
Replication of the *Igh* locus in *Pax5* ^−^^/−^ *Rag2* ^−^^/−^ uncommitted pro-B cells (129/Sv). The panels containing experimental data (A–D) appear discontinuous because independent experiments were performed for each restriction fragment. (A) The top portion of this panel shows the schematics of the four restriction fragments depicted in Figure 1A (shown to scale). Red and green bars indicate the positions of the initiation and termination events detected by SMARD. Numerals indicate the normalized frequency of the events scored in each population of restriction fragments (this value represents an underestimate of the actual number of events taking place during every replication cycle, a fact that is taken in consideration by our mathematical model; see Materials and Methods). These events are organized from top to bottom by increasing size of the labeled region. Some bars are shown adjacent to each other to emphasize that they occupy non-overlapping locations; this does not mean that the events occurred on the same DNA molecule. The midpoint on each bar is marked by black symbols to indicate the most likely position of an active origin or fork collision. Gray arrows show the predominant directions of replication fork movement across the region. (B, C) Distribution of the replication forks moving leftward and rightward, from SMARD (empty symbols), and from the fit (line). Each value indicates the number of replication forks per kb (fork density) detected in the population of double-labeled DNA molecules after averaging the result over intervals of defined size (10 kb for the *Pme*I fragments, 5 kb for *Pac*I#3 and *Swa*I#2). Trends in fork direction can be detected by comparing, for individual fragments, the densities shown in (B) and (C). The densities of different fragments cannot be compared directly because this value is a function of the replication time, which varies for each fragment (Table S1, Column i). Error bars were calculated as described in Materials and Methods. (D) IdU content of the population of double-labeled DNA molecules averaged over intervals of defined size (see panel B), obtained experimentally by SMARD (empty symbols), and from the fit (line). For each of the four restriction fragments, individual data points indicate the fraction of double-labeled molecules substituted with IdU within that interval. Since each fragment was studied individually, discontinuities are visible. However, adjacent fragments show similar trends in the shape of their graphs (increasing or decreasing), allowing us to deduce the position of the regions replicating first (peaks) and last (valleys). Error bars were calculated as described in Materials and Methods. (E) Distribution of the firing rate of origins across the *Igh* locus (blue line) and at adjacent genomic locations (red and blue arrows). Changes in the firing rate are shown continuously along the 1.4 megabase region spanned by the four restriction fragments. The firing rate is expressed in initiations/kb/minute to indicate that during each minute of the S phase, within a specific 1 kb section of the locus, a certain number of origins will fire in a population of 10^−6^ unreplicated DNA molecules (see also Table 1). This figure also shows the efficiency of origin firing within specific sections of the *Igh* locus (gray dashed lines), with gray numerals indicating the number of initiation events occurring in each region, per allele, per S phase (IAS). (F) Curves showing extent of DNA replication at different times in S phase for the various parts of the *Igh* locus (10%, 50%, and 90% replicated).

In the double-labeled DNA molecules, IdU-CldU transitions mark the positions of replication forks at the time of the label switch. Initiation events appear as IdU-labeled regions surrounded by CldU, while fork collisions display the complementary pattern (e.g., Figure 1C–D). Figure 2A summarizes the location of each initiation event (red bars) and fork collision (green bars) detected in 158 individual restriction fragments. The normalized frequency of the events across the four fragments is also indicated (expressed as number of events scored per 100 double-labeled DNA molecules, per 100 kb). The values before normalization are presented in Table S1. Note that initiation events and fork collisions spanning adjacent restriction fragments are not shown in the figure since these events are scored as simple IdU-CldU transitions (a fact that is taken into consideration by our mathematical model and in Figure S2C). Overall, these results indicate that initiation events are more frequent near the D_H_-J_H_ and middle-V_H_ gene families than in other parts of the locus. In these regions, the presence of non-overlapping events implies the existence of clusters of active origins that are reminiscent of initiation zone [23],[24]. In contrast, fork collisions are more frequent between the origin clusters (termination regions in Figure 2A).

It is important to point out that SMARD experiments are designed to detect only a fraction of the active origins (the number of double-labeled DNA molecules required to reach mapping saturation increases with the number of potential origins). Moreover, reaching mapping saturation is increasingly unlikely where origin efficiency is low, and near the end of the restriction fragments. Thus, Figures 2A and S2 are expected to largely underestimate the actual density of origins along the locus (see definition in Table 1). Even so, the distance between the midpoints of initiation event within the D_H_-J_H_ region suggests an origin density higher than one potential origin per 10 kb (Figure S2E, *Pac*I#3). In Figure 2A, origin density appears lower near the middle-V_H_ region genes. However, within this section of the locus, the midpoints of initiation events overlap only rarely, indicating that that origin mapping is far from saturation (Figure S2E, *Pme*I#5). Lack of saturation is even more pronounced across the proximal-V_H_ region (none of the detected initiation events overlap; Figure 2A, *Pme*I#4). We conclude that potential origins of replication are likely to be present at a relatively high density across most of the *Igh* locus.

Since cells grow asynchronously during DNA labeling, the IdU-CldU transitions depict all stages of DNA replication, reflecting the dynamics present at the steady-state of growth (right panel in Figure S1) [2]. This means that the population of double-labeled DNA molecules can be used to determine the steady-state distribution of replication forks across the *Igh* locus (arrowheads in Figure 2B–C), and the average number of forks participating in the replication of each restriction fragment (Table S1, column l). Similarly, the temporal order of replication can be obtained from the level of IdU substitution of these molecules, with peaks marking the regions that replicate first and valleys marking the regions that replicate last (Figure 2D). Overall, these results indicate that, in uncommitted pro-B cells, DNA replication tends to begin near the D_H_-J_H_ and middle-V_H_ gene families, as well as at origins located 3′ and 5′ of the *Igh* locus. From there, replication forks proceed to replicate the locus until they collide with an oppositely moving fork (predominantly within the termination regions).

### The Replication Dynamics of the *Igh* Locus Are Consistent with the Stochastic Firing of Origins and with Variations of Their Firing Rate along the Genome

The data presented above show that the replication of the *Igh* locus follows a precise temporal program. However, this program represents a population average. At the level of individual DNA molecules, initiation events do not seem to occur in any particular order, with different origins firing in different molecules. Initiation events were also detected within the portions of the *Igh* locus that replicate last (e.g., the termination region within *Pme*I#4 and the region between Cδ and Cγ3; Figure 2A), taking place as the corresponding molecules were at various stages of their replication. Such events are not easily explained by deterministic models of origin activation. A domino activation of origin firing [13] also seems unlikely. The initiation events detected in the population of double-labeled DNA molecules are rarely associated with externally generated replication forks, and when such forks are present their location is tens to hundreds of kilobases from the firing origins (e.g., 40 and 100 kb in the example shown in Figure S2A). Hence, it is possible that *Igh* origins fire according to stochastic dynamics.

To determine whether the results obtained by SMARD can be quantitatively explained by stochastic origin firing (see definition in Table 1), we used a mathematical formalism and a simulation procedure that we recently developed for this purpose [18]. As briefly described in Materials and Methods, this procedure allows us to fit many of the data collected by SMARD (namely, the distribution of the replication forks, Figure 2B–C, the temporal order of replication, Figure 2D, and the replication time of the restriction fragments, *Tr*, Figure S2B) to computer-generated curves calculated from a series of rate equations.

In the simplest possible scenario, the curves were calculated assuming that bidirectional origins of replication fire stochastically throughout the *Igh* locus and the S phase (see Table1 for a precise definition), generating forks that move at a constant speed. In these calculations, the rate of origin firing was the only parameter allowed to vary freely across the *Igh* locus, while remaining constant in time (meaning that, within a genomic region, initiation events continue to occur at the same rate from the beginning of S phase until the region is replicated in the entire population of cells). It is important to point out that the rigid constrains imposed by this scenario do not accurately reflect physiological conditions. For example, it is known that modest changes in the speed of replication forks can indeed occur along the genome and during the S phase [25]. However, by limiting the number of free variables, this scenario allows us test the stochastic firing hypothesis more stringently.

Strikingly, we found that this simple scenario is sufficient to reproduce all experimental data sets collected by SMARD (Figures 2B–D and S2B–C). This scenario can even reproduce data sets that were not used during the fitting procedure (such as the location of initiation events and fork collisions, the number of molecules containing such events, the average number of events per molecules, and the average speed of replication forks). Since the fit was performed simultaneously for all restriction fragments, the presence of local discrepancies is not particularly surprising (e.g., *PmeI* #4 in Figures 2D and S2B). This is likely to reflect the constrains imposed by this scenario (e.g., within some portions of the locus fork speed may deviate from the average and origin density may also vary). Nevertheless, the high quality of the fit (reduced chi-square, 1.18) indicates a close match between calculated and experimental data sets. Hence, the results collected by SMARD are fully compatible with the stochastic firing of origins throughout the *Igh* locus.

In addition to the changes in firing rate along the locus, scenarios involving a larger number of variables were also considered (e.g., allowing for a variable speed of replication forks or for changes in the rate of origin firing during the S phase). In principle, changing these parameters could have a major effect on the replication dynamics of the *Igh* locus. However, these scenarios improved the quality of the fit only marginally (M.G.G., J.B., and P.N., unpublished observation). This means that variations in parameters other than the firing rate of origins along the genome, while possible, have a limited impact on the replication dynamics and the temporal order of replication of the *Igh* locus in uncommitted pro-B cells.

### The Temporal Order of Replication Is Determined by Wide Differences in the Firing Rate of Origins across Large Sections of the *Igh* Locus

The high quality of the fit calculated above indicates that the computer-generated data set provides a good approximation of the firing rate of origins across the *Igh* locus. Figures 2E and S2D show that the firing rate is very low throughout most of the locus (4.5×10^−6^ initiation events per kb per minute). Two exceptions are the D_H_-J_H_ region (55±13.2 kb in size) and of the middle-V_H_ region (281±43.1 kb in size), where the firing rate is up to 77-fold higher. This variation implies that origins located in different parts of the *Igh* locus have a very different tendency to fire.

Another striking feature of these results is that the firing rate changes abruptly at a few specific locations (transitions), while remaining virtually uniform across large sections of the locus (plateaus). Attempts at fitting bell-shaped curves (which lack both plateaus and sharp transitions) produced fits of lower quality compared to box-shaped curves of the kind shown in Figure 2E (M.G.G., J.B., and P.N., unpublished observation). Therefore, the *Igh* locus appears to be organized in precisely defined domains where origins have a similar rate of firing (suggesting a significant degree of coordination among the origins of individual domains). We conclude that the temporal order of replication is a consequence of the combined effect of domain sizes and firing rates.

Equation 3, in Materials and Methods, also allows us to determine the efficiency of origin firing for specific sections of the *Igh* locus, according to the definition provided in Table 1. The values obtained for the D_H_-J_H_ and middle-V_H_ regions are only 1.1 and 1.5 initiation events, per allele, per S phase (IAS, gray dashed lines in Figure 2E). These regions are known to contain multiple origins of replication (Figure 2A). Hence, most of the origins remain silent during each cycle of replication (firing is inefficient). Notably, the portion of *Igh* locus spanning the proximal-V_H_ genes produces 0.4 IAS even if its firing rate is about 2 orders of magnitude lower than the D_H_-J_H_ and middle-V_H_ regions. The nonlinear relationship between firing rate and origin efficiency can be explained by the fact that the proximal-V_H_ genes occupy a very large genomic region and tend to replicate after adjacent portions of the locus (Figure 2D). This provides more time for the origins therein located to fire. Hence, efficiency is not an intrinsic property of individual origins, but rather the result of the distribution of the firing rate throughout the *Igh* locus.

The computer-generated data set allows us to draw a few additional conclusions. Although *Igh* origins fire inefficiently, they are responsible for the replication of 87% of the locus, with only minor contributions from external origins (Figure S2F). This means that inefficient origin firing extends beyond the margins of the locus into the surrounding regions. In addition, various portions of the locus have a very high probability of being replicated by forks moving in one particular direction (peaks and valleys in Figure S2G). Since the molecules analyzed in this experiment originated from both *Igh* alleles, the strong bias in fork direction indicates that both alleles follow a similar replication program. Finally, we calculate that it takes approximately 4 h to complete the replication of this portion of the genome in 100% of the cell population (Figure 2F). Hence, these experiments provide a complete description of DNA replication, within the *Igh* locus, over a broad portion of the S phase.

### The Firing Rate Is Regulated Independently in Different Domains of the *Igh* Locus

Bone marrow pro-B cells from *Pax5* ^−*/*−^ mice are blocked at the uncommitted stage of differentiation but, unlike *Pax5* ^−*/*−^ *Rag2* ^−*/*−^ cells, they can undergo D_H_-J_H_ recombination. The resulting loss of various sections of the D_H_-J_H_ origin cluster (the ∼55±13.2 kb region containing the origins with the highest firing rate; Figure 2E) provides us with a tool to study how the firing rate is regulated. Specifically, we can use ex vivo propagation to obtain clonal populations of cells carrying particular D_H_-J_H_ deletions. Here, we studied a heterozygous pro-B cell clone that carries a 65 kb deletion on its 129/Sv allele and a 25 kb deletion on the C57BL/6 allele. The different sizes of the resulting restriction fragments allowed us to use SMARD to investigate the effect of each deletion on DNA replication.

**Figure 3:**
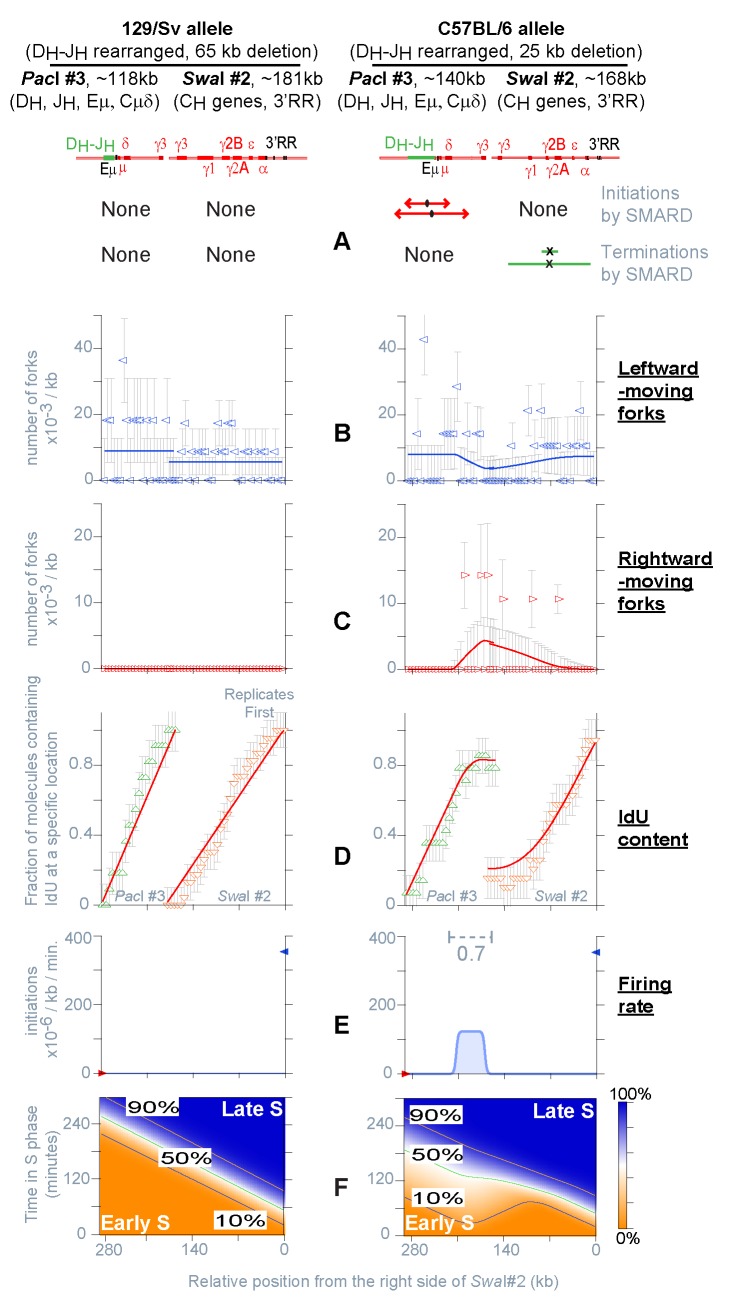
Replication of the *Igh* locus in a D_H_-J_H_ rearranged clonal population of *Pax5* ^−^^/−^ uncommitted pro-B cells (129/Sv-C57BL/6). The results for each allele are shown separately. Only two restriction fragments were analyzed (*PacI* #3 and *SwaI* #2). (A–F) Summary of the results obtained by SMARD from the analysis of 1,443 hybridization signals, yielding 532 fully substituted DNA molecules, 313 meeting the standards of measurement, for a total of 67 double-labeled molecules (see Table S1 for details). The results of the fitting procedure are shown as described for Figure 2.

Consistent with the loss of the entire D_H_-J_H_ origin cluster, the 65 kb deletion reduced the number of initiation events occurring within fragment *PacI#3* to an undetectable level (left portion of Figure 3A and Figure S3B). The direction of replication fork movement (Figure 3B–C) and the uniform slope of the temporal order of replication (Figure 3D) indicate the passive replication of the region by forks originating 3′ of the *Igh* locus. This is similar to results previously obtained in non-B cells, where the *Igh* locus is part of a replication timing transition region [2]. Besides for the loss of the D_H_-J_H_ origins, the rate of origin firing in adjacent portions of the locus remains unchanged (compare *PacI#3* and *SwaI#2* in Figures 2E and 3A). This rate is very low compared to the origins located at 3′ of the *Igh* locus. Hence, even if the 5′-end of *PacI#3* now replicates 3 h later compared to unrearranged pro-B cells (compare Figure 3F at 280 kb to the corresponding portion of Figure 2F), this delay is not sufficient to produce large numbers of initiation events within *PacI#3* and *SwaI#2* (infrequent initiation events may still occur within this portion of the *Igh* locus but their visualization would require the analysis of a much larger sample of double-labeled DNA molecules).

In contrast, the 25 kb deletion removes less than half of the D_H_-J_H_ origin cluster. On this allele, initiation events and fork collisions continue to occur at the same locations described for *Igh* locus in germline configuration (compare the right portion of Figure 3A with Figure 2A). However, there is a strong right-to-left bias in the direction of fork movement (Figure 3B–C), and the IdU content shows a steady decrease in the same direction with an inflection point at the site of the remaining D_H_-J_H_ origins (Figure 3D). These factors indicate the passive replication of the region in a fraction of the cell population. Accordingly, there is a 40% reduction in the number of initiation events occurring within this section of the locus during each S phase (0.7 IAS, Figure 3E), compared to the level detected in unrearranged pro-B cells (1.1 IAS, Figure 2E). These results indicate that the decrease in initiation events is proportional to the reduction in size of the D_H_-J_H_ origin cluster. Hence, the firing rate of the remaining *Igh* origins and the location of the firing rate transitions are unaffected by the 25 kb deletion (Figure 3E). This is reminiscent of results obtained by studying deletions of the DHFR locus by 2D-gel electrophoresis (although in that case conclusions were based on measurement of origin efficiency, which is context dependent, and not of firing rate) [26],[27]. We conclude that firing rate of origins is regulated independently in different sections of the locus and that origin activity at one location is not affected by the presence, or absence, of neighboring origins.

### B Cell Commitment Alters the Firing Rate of Origins throughout the *Igh* Locus with the Largest Changes Occurring in Domains that Contain Pax5 Binding Sites

**Figure 4:**
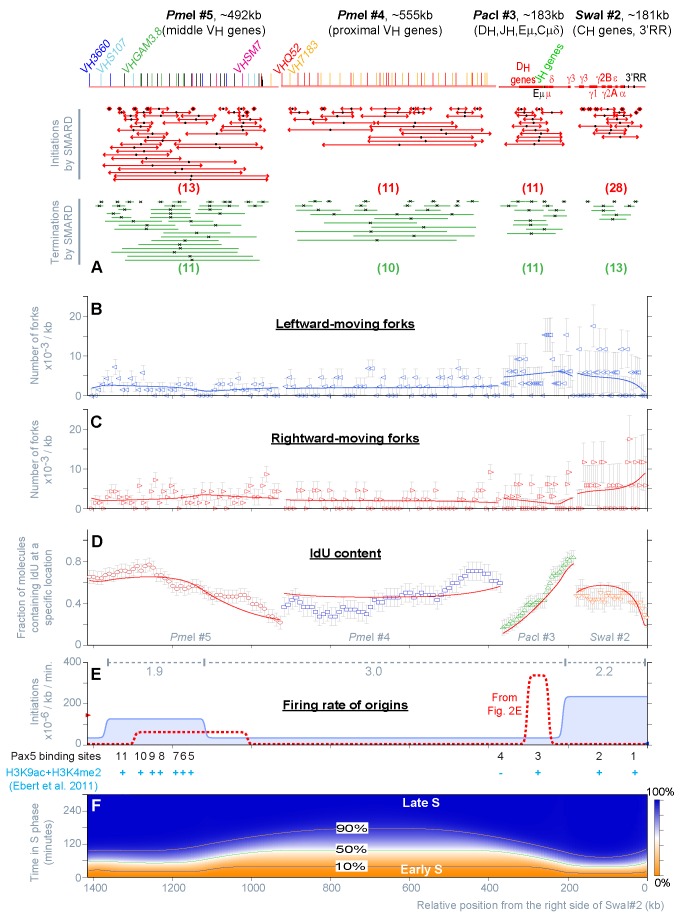
Replication of the *Igh* locus in *Rag2* ^−^^/−^ committed pro-B cells (129/Sv). (A–F) Summary of the results obtained by SMARD from the analysis of 973 fully substituted DNA molecules, 683 meeting the standards of measurement, for a total of 213 double-labeled molecules (see Table S1 for details). The results of the fitting procedure are shown as described for Figure 2. The locations of Pax5 binding sites in *Rag2* ^−*/*−^ committed pro-B cells, as well as some chromatin modifications, are also indicated in panel E (see Materials and Methods for additional details).

In order to study how the replication of the *Igh* locus changes during cell differentiation, we isolated B cell progenitors from the bone marrow of a *Rag2* ^−*/*−^ mouse (129/Vs.). These cells efficiently undergo B cell commitment but maintain the *Igh* locus in germline configuration, which prevents them from developing any further. In these cells, initiation events and fork collisions are distributed across most of the *Igh* locus (Figure 4A). Only a few of these initiation events are centered at the same genomic location (Figure S4), indicating that the experiment is far from mapping saturation (leading to an underestimate of origin density). This suggests that potentially active origins are present at high density across most of the *Igh* locus, perhaps every 10–20 kb, although larger gaps may exist at a few locations. Despite the widespread activation of origins, it is still possible to distinguish portions of the locus where replication forks have a preferred direction of movement (e.g., *Pac*I#3; Figure 4B–C) and regions that replicate first and last (Figure 4D). Thus, our results indicate that the level of origin activity is not uniform across the *Igh* locus and point to major differences in DNA replication between committed and uncommitted pro-B cells.

Once more, we found that the data obtained by SMARD are fully compatible with the stochastic firing of origins (reduced chi-square, 1.03; Figure S4C). The nearly perfect fit confirms that the temporal order of replication is mostly determined by the regulation of a single variable along the locus (the firing rate of origins). Computer calculations also show that the rate of origin firing is not uniform (blue line in Figure 4E). A comparison with the results obtained for uncommitted pro-B cells (red dotted line) reveals that B cell commitment is accompanied by changes in the rate of origin firing across most of the *Igh* locus. However, the largest changes (whether positive or negative) occur in regions where a previous genomic screening identified the presence of Pax5 binding sites in committed pro-B cells [28]. For example, the firing rate increases nearly 50-fold across the CH-3′RR region (∼216 kb in size and containing two Pax5 binding sites) and decreases 10-fold throughout the DH-JH origin cluster (∼55 kb in size and containing one Pax5 binding site). These results indicate that the rate of origin firing is the parameter that is being regulated during cell differentiation. In addition, they suggest that Pax5 participates in regulating the firing rate of origins during B cell commitment (although the number of known binding sites for this factor is far smaller than the number of potential origins affected by its expression).

As a result of B cell commitment, changes in origin efficiency were also observed throughout the *Igh* locus (compare gray dotted lines in Figures 2E and 4E). However, the overall efficiency of origins across the 1.4 Mb region increases only from 3.2 IAS in uncommitted pro-B cells (Figure S2D) to 7.4 IAS in uncommitted pro-Bs (Figure S4C). Thus, the firing rate can increase by 1–2 orders of magnitude across most of the *Igh* locus but produce variations of only 2.3-fold in the total number of initiation events (and replication complexes) involved in the replication of the locus. Given the high density of potential origins throughout the *Igh* locus, these results also suggest that the firing efficiency of individual origins is below 10%. Therefore, following B cell commitment, origins continue to fire inefficiently even if the firing rate increases across most of the *Igh* locus.

### Inducing Pax5ER Increases the Firing Rate of Origins within the C_H_-3′RR Region and Changes the Temporal Order of Replication

**Figure 5:**
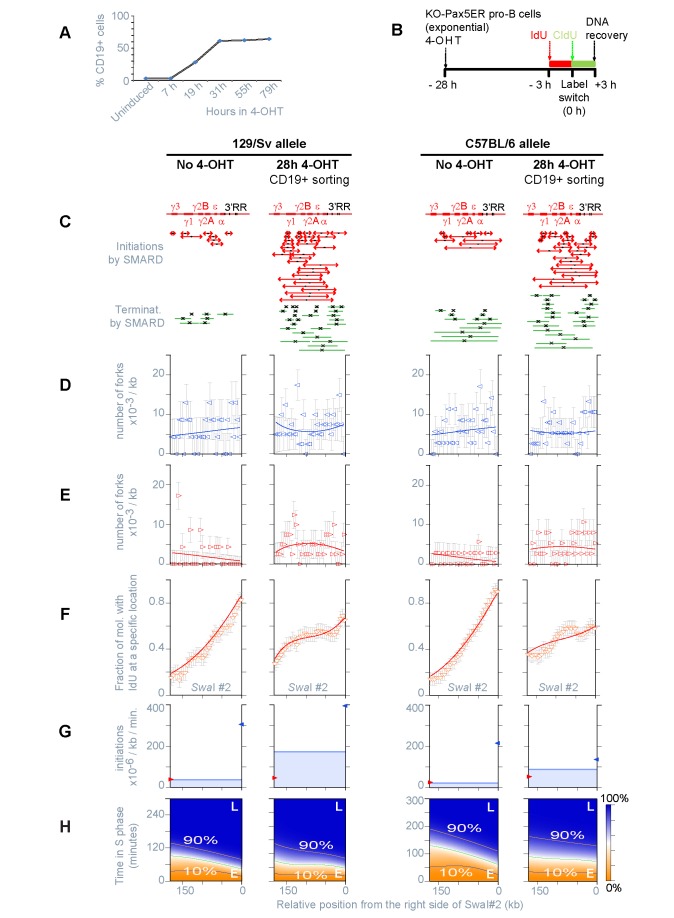
Replication of the C_H_-3′RR region in KO-Pax5ER pro-B cells (129/Sv-C57BL/6). (A) Kinetics of expression of CD19 after 4-OHT induction. The number of CD19^+^ cells reaches a plateau at about 30 h. (B) Scheme of Pax5ER induction and DNA labeling used for the SMARD. (C–H) Summary of the SMARD experiments performed to study the replication of C_H_-3′RR region before and after induction with 4-OHT (from the analysis of 1,832 fully substituted DNA molecules, 1,445 meeting the standards of measurement, for a total of 339 double-labeled molecules; Table S1). Each experiment is presented in a different column. Results for the two *Igh* alleles are shown separately (129/Sv, C57BL/6). The results of the fitting procedure are shown as described for Figure 2.

Reconstituting Pax5 expression in *Pax5* ^−*/*−^ pro-B cells induces B-cell commitment [20],[29] and can be used to study the role of this protein in origin regulation. For this purpose, we transduced bone marrow pro-B cells from a *Pax5* ^−*/*−^ mouse (129/Sv-C57BL/6) with a retroviral vector containing the expression cassette *Pax5ER-IRES-GFP* [30],[31]. We then sorted and expanded the GFP^+^ cells to obtain a polyclonal population expressing Pax5ER (KO-Pax5ER pro-B cells; Figure S5A–B). This protein is the fusion product of Pax5 and the hormone-binding domain of the estrogen receptor, which becomes biologically active in the presence of 4-hydroxy-tamoxifen (4-OHT). In KO-Pax5ER pro-B cells, 4-OHT is able to induce commitment-specific changes in the methylation of the 3′RR DNA, indicating that Pax5ER is able to interact with at least one of the Pax5 binding sites of the C_H_-3′RR region [32]. In KO-Pax5ER pro-B cells, the occurrence of B-cell commitment was monitored by the Pax5-dependent expression of the cell-surface-marker CD19. Before the addition of 4-OHT, we consistently found that only 4% of KO-Pax5ER pro-B cells are CD19^+^ (Figures 5A and S5C). However, 65%–90% of cells become CD19^+^ after the addition of 4-OHT. Thus, in our inducible system, the activity of Pax5ER is modest in the absence of 4-OHT but increases dramatically after induction, leading to B cell commitment.

Can the induction of Pax5ER increase the firing rate of origins within the C_H_-3′RR region? To answer this question, we studied KO-Pax5ER pro-B cells before and after induction with 4-OHT for 28 h (see labeling scheme in Figure 5B). This induction time was chosen because it allows enough time for the synthesis and turnover of the gene products regulated by Pax5 and for cells to become fully committed to the B lineage [30]. In the absence of 4-OHT, we detected a limited number of initiation events within the C_H_-3′RR region, which is consistent with the modest activation of Pax5ER described above (first and third column in Figure 5C). However, this portion of the locus continues to be passively replicated in the majority of cells, as indicated by the replication fork distribution and the temporal order of replication (Figures 5D–F and S5H–I). In contrast, 4-OHT induction profoundly alters all aspects of DNA replication within the C_H_-3′RR region, suggesting a strong increase in origin activity (second and fourth column in Figures 5C–F and S5H–I). Numerical calculations confirm that the firing rate of origins reaches the level detected in committed pro-B cells (compare the 129/Sv results in Figures 5G and 4E). Hence, inducing B cell commitment in vitro produces changes in the firing rate of *Igh* origins that are similar to those observed in pro-B cells isolated from mice.

Additional experiments confirmed that these effects are specific. For example, the firing rate of C_H_-3′RR origins does not increase when 4-OHT is provided to uncommitted pro-B cells that do not express Pax5ER (Table S1). In addition, the induction of Pax5ER in KO-Pax5ER pro-B cells does not affect origin activity broadly and non-specifically. For example, we did not observe significant changes in cell proliferation and cell-cycle profile (Figure S5L) or the appearance of markers of DNA damage and DNA damage checkpoint activation (Figure S5M). We conclude that the change in origin activity observed in KO-Pax5ER pro-B cells results from the activation of Pax5ER and the induction of B cell commitment.

### The Regulation of Firing Rate of Origins Does Not Require Changes in Gene Expression and Takes Place in Late G1

B cell commitment involves changes in the expression of hundreds of tissue-specific genes [33], raising the question of whether Pax5 is directly responsible for regulating the firing rate of origins. After the addition of 4-OHT, Pax5ER requires only a few minutes to translocate from the cytoplasm to the nucleus, where it changes the expression of its target genes over a period of several hours [30]. We suspected that the firing rate would change very slowly if its regulation depended on the activation or silencing of these genes. Therefore, we decided to determine the speed at which the firing rate increases within the C_H_-3′RR region following the addition of 4-OHT to KO-Pax5ER pro-B cells.

**Figure 6:**
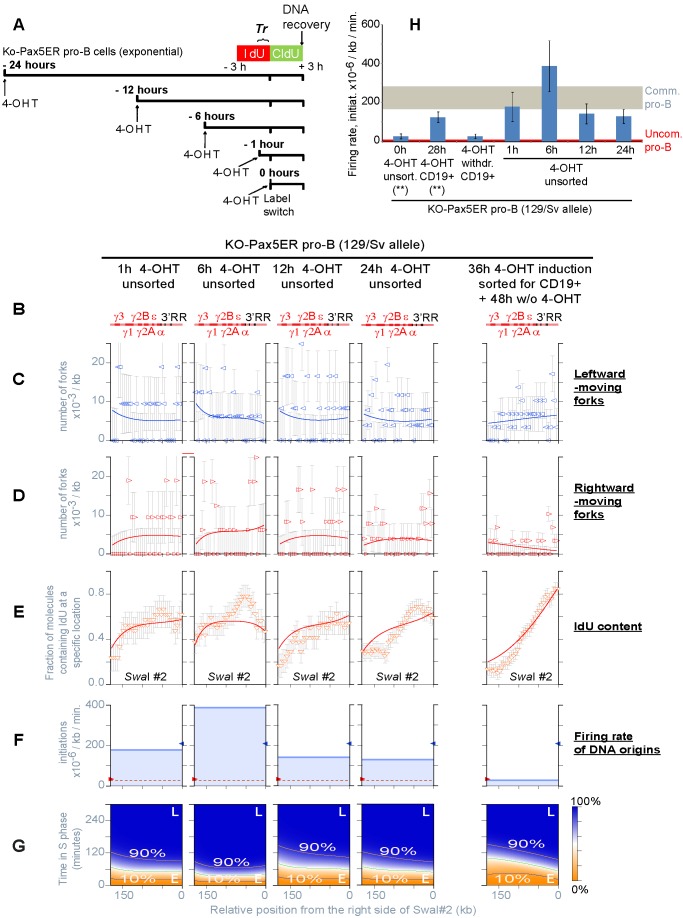
Kinetics of the changes in origin activity induced by 4-OHT (and by 4-OHT withdrawal) within the C_H_-3′RR region in KO-Pax5ER pro-B cells. (A) Labeling scheme utilized for the time-course experiment in asynchronously growing cells. *Tr* indicates the time interval during which the double-labeled DNA molecules start replicating (see Table S1 for details). (B–G) Summary of the results obtained by SMARD from the analysis of 1,013 fully substituted DNA molecules, 818 meeting the standards of measurement, for a total of 202 double-labeled molecules (see Table S1 for details). The results of the fitting procedure applied to all experimental data sets obtained for the 129/Sv allele are shown as described for Figure 2. (H) Firing rate of origins calculated from the simultaneous fit of all experimental data sets obtained for the 129/Sv allele, including those presented in Figure 5 (**).

The labeling scheme for the time-course experiment is presented in Figure 6A. Our experimental design takes advantage of the fact that SMARD detects the occurrence of initiation events in the population double-labeled DNA molecules. These molecules begin their replication during a precise interval of time *Tr* that preceded the label switch, meaning that they originate from cells that entered S phase sometimes before each molecule started replicating [2]. As a result, we can study how DNA replication initiates, progresses, and terminates across the genomic region of interest, knowing that these events occurred in cells that crossed the G1/S transition during a specific window of time from the addition of 4-OHT (this procedure also avoids cell synchronization and experimental manipulations that may generate experimental artifacts).

Surprisingly, we found that the replication of the C_H_-3′RR region changes very rapidly after the addition of 4-OHT. For all induction times between 1 and 24 h, the distributions of replication forks and the temporal orders of replication are similar to the experiment performed at 28 h (compare Figure 6B–E with Figure 5D–F). To accurately determine how the firing rate changes over time, we simultaneously fit all experimental data sets obtained for the 129/Sv allele (including those presented in Figure 5). This resulted in a reduced chi-square of 1.02 (Figure S6A–C). As summarized in Figure 6H, the firing rate of the C_H_-3′RR origins reaches a level comparable to committed pro-B cells within the first hour of induction. Given that this induction time is similar to the replication time of the restriction fragment (approximately 1 h; Table S1), we conclude that the firing rate of origins began to increase within minutes after the addition of 4-OHT. This rapidity implies that the changes in firing rate do not depend on modifications in gene expression and are likely to be caused by a different mechanism (e.g., transcription, chromatin remodeling, etc.).

But how is the regulation of the firing rate of origins achieved during cell differentiation? It is important to note that the population of double-labeled DNA molecules originates from cells that are in S phase at the time of the label switch, having crossed the G1/S transition 0 to 80′ before each molecule started replicating (Figure S7). This means that the 7-fold increase in the firing rate observed after 1 h of induction (Figure 6H) takes place in cells that were first exposed to 4-OHT at a time in the cell cycle close to the G1/S transition (between 60′ before G1/S and 80′ into S phase; Figure S7). We know that, in mammalian cells, the replicative helicase is loaded between the end of mitosis and early G1, completing the process of origin licensing and the formation of pre-replication complexes (pre-RCs) as reviewed in [34]. We can therefore conclude that Pax5 controls the rate of origin firing, and the temporal order of replication, by affecting a stage in origin activation downstream of pre-RC assembly. A corollary to this conclusion is that pre-RCs can form across the C_H_-3′RR region even in the absence of 4-OHT. Therefore, pre-RCs can form efficiently even in genomic regions characterized by a low firing rate of origins.

### Events Prior to the Assembly of Pre-RCs Have Only Minor Effects on the Firing Rate of Origins and the Temporal Order of Replication

Are the effects of Pax5ER induction limited to the late stages of origin activation? Figure 6H shows that the firing rate of the C_H_-3′RR origins changes over time, peaking at 6 h induction and returning to the level of committed-pro-B cells thereafter (at 12, 24, and 28 h). We know that the cell cycle of KO-Pax5ER pro-B cells is approximately 14 h and that their G1 lasts about 6 h (A.D. and P.N., unpublished observation). This means that the 14-fold increase in firing rate that we observe during the first 6 h of induction is linked to events taking place during the preceding G1 (mostly after the assembly of the pre-RCs). In contrast, the firing rate measured at 12 h of induction is the result of events taking place not only after pre-RC assembly but also before (during the previous M, G2, and S phases). Since this value is higher than the pre-induction level but 63% lower than the firing rate measured at 6 h (Figure 6H), we conclude that some inhibition of the firing rate takes place during the previous M, G2, and S phases. However, this inhibition is insufficient to fully compensate for the increase in firing rate that takes place during G1. We conclude that the firing rate of the C_H_-3′RR origins is affected by the induction of Pax5ER at various stages of the cell cycle but is dominated by events that occur after pre-RC assembly.

### The Activity of Pax5 Is Continuously Required to Maintain a High Rate of Origin Firing within the C_H_-3′RR Region

Are the changes in firing rate induced by Pax5 stable? Or do they need to be reestablished at every cell division? To address this question, we took advantage of the fact that the activation of Pax5ER can be slowly reversed by OHT withdrawal. We first grew KO-Pax5ER pro-B cells in the presence of 4-OHT for 36 h to induce full B cell commitment and to increase the rate of origin firing within the C_H_-3′RR region. We then isolated the population of committed pro-B cells (CD19^+^ fraction), expanding it in the absence of 4-OHT for 48 h before labeling the replicating DNA and performing SMARD. Results indicate that, following 4-OHT withdrawal, origin activity and the temporal order of replication revert to pre-induction levels (right portion of Figure 6 and Figure S6). Therefore, the activity of Pax5 is continuously required to maintain high rates of origin firing within the C_H_-3′RR region (these changes are not epigenetically inherited). This also means that a low firing rate represents the default state for these *Igh* origins, an observation that is consistent with the low level of origin activity observed in all types of non-B cells examined to date [2].

## Discussion

In this study, we provide a model of origin activation that can explain multiple parameters of DNA replication initiation, progression, and termination throughout a significant portion of the S phase. Parameters, such as the location of initiation events and fork collisions, the efficiency of origin firing, the number and distribution of replication forks, the timing of origin firing, and the temporal order of replication can be quantitatively explained by the interplay between two independent variables: the speed of replication forks and the firing rate of origins. Only the latter varies extensively along the locus representing the dominant variable. The firing rate of origins is also the main variable that is being regulated during cell differentiation.

For each individual experiment, both independent variables can be considered constant in time (an approximation that is sufficiently accurate to describe the events taking place within the *Igh* locus during the first few hours of S phase but does not exclude the possibility of variations over the entire S phase). Other aspects of DNA replication behave as dependent variables that do not require dedicated regulatory pathways. This means that the extensive literature concerning the regulation of origin efficiency and the timing of origin firing can be reexamined in terms of changes in the firing rate of origins. For example, the apparent discrepancies mentioned in the introduction regarding some earlier studies [2],[3],[5] could be reconciled by changes in the firing rate of origins during cell differentiation. Hence, our model can provide a simple conceptual framework to explain the dynamics of DNA replication at a specific mammalian locus.

Our study suggests that potentially active origins are present at high density throughout the *Igh* locus in both committed and uncommitted pro-B cells (Figures 2A, 4A, S2E, S4D). Active origins were detected even within portions of the locus that replicate last (e.g., PmeI#4 in Figure 2). This interpretation is consistent with recent observations showing the presence of active origins of replication in portions of the human IGH locus previously thought to contain only silent ones [13],[35]. However, based on the analysis of a larger set of parameters of DNA replication (representing all stages of replication), we showed that the initiation events detected experimentally can be explained by an entirely stochastic mechanisms of activation (meaning that origin firing can occur in any unreplicated portion of the *Igh* locus, at any time during the S phase).

Our study also suggests a significant degree of coordination in the level of origin activity across large sections of the *Igh* locus (tens to hundreds of kilobases in size). Within these domains, the firing rate appears to be relatively uniform and is modulated synchronously, over a broad range, as a result of changes in cell differentiation. The temporal order of replication is determined by the combined effect of variable domain sizes and firing rates. Consistent with studies performed at other mammalian loci [26], we showed that groups of origins can be deleted from the *Igh* locus without significantly affecting the firing rate of the remaining origins, or the location of the firing rate transitions (these two qualities of chromosomal domains are not significantly affected by the activity of individual origins of replication). In contrast, clustering of yeast origins with similar characteristics has been observed in various studies, but the firing rate of each origin is different and appears to be individually regulated [15],[36],[37]. Hence, our results point to significant differences in the way origin activity is regulated in the two organisms, perhaps reflecting a role of domain boundaries in the modulation of mammalian DNA replication (see below) [38].

During B cell commitment, the largest changes in firing rate occur in *Igh* domains that contain Pax5 binding sites and depend on the continuous expression of this protein. This suggests that cell-specific factors participate in controlling the temporal order of replication by regulating the firing rate of origins within specific chromosomal domains. Pax5 modifies the firing rate of origins using at least two separate mechanisms that operate at different times during the cell cycle. One mechanism acts upstream of pre-RC assembly. It produces a stable decrease in the peak rate of origin firing across the C_H_-3′RR region, suggesting it affects a limiting step in origin activation (perhaps the formation of pre-RCs). However, the limited magnitude of the change means that this mechanism can only marginally affect the temporal order of replication. In contrast, within the C_H_-3′RR region, the rate of origin firing is mostly determined by events occurring in late G1/early S (after the loading of ORC and MCM proteins). This second mechanism produces a rapid increase in the firing rate of origins that continues for a few hours after the induction of Pax5ER (cells need to be exposed to Pax5 for most of G1 in order to reach the maximum level of origin activity). These results are consistent with recent observations suggesting that temporal order of replication is strongly affected by events downstream of the assembly of pre-RCs [39],[40].

Given that Pax5 affects origin activity in multiple ways, dissecting its activity will require additional studies. However, it is important to point out that chromosomal loops comparable to the C_H_-3′RR, D_H_-J_H_, proximal-V_H_, and middle-V_H_ domains have been recently identified by 4C and 3C assays in C57BL/6 committed pro-B cells [41]. This suggests that the sharp transitions in firing rate detected in our study may occur at the bases of chromosomal loops; with plateaus making the body of each loop (adjacent loops with a similar firing rate may not be distinguishable). Interestingly, the loop organization of the *Igh* locus can change during B cell differentiation [42]. In addition, Pax5 has been shown to alter the chromosomal architecture of various portions of the locus, inducing locus contraction [43], and promoting chromatin looping within the C_H_-3′RR region [44]. Hence, it seems plausible that chromosomal topology and domain boundaries play a role in modulating the firing rate of origins within the *Igh* locus. In this context, the role of Pax5 could be helping to form new chromosomal loops or altering the topology of preexisting ones. Similar mechanisms could provide a way to affect the activity of multiple origins of replication across large sections of the genome (perhaps by changing their accessibility to rate-limiting factors or by facilitating DNA unwinding at origins). In fact, preliminary evidence indicates that the deletion of short regulatory elements can affect the firing rate of origins across entire *Igh* domains (P.N., unpublished result).

One of the appeals of the model of origin regulation presented above is that physiological variations in a range of parameters of DNA replication can automatically be compensated without necessarily requiring the intervention of checkpoints. For example, a localized decrease in replication fork progression will provide more time for origins to fire, resulting in the activation of additional origins (proportionally to their firing rate). This can limit the probability that sections of the genome will remain unreplicated for extended periods of time. A similar compensatory mechanism can operate at the genomic level as a result of cell-to-cell variations in origin licensing, or due to factors that are rate-limiting for origin firing [45]. Even in the presence of a drastic reduction in the global level of origin activity (e.g., following the activation of DNA damage checkpoints), residual initiation events are expected to continue to occur according to the differential in firing rate of origins in the various portions of the genome (therefore preserving the temporal order of replication). These predictions are consistent with experimental observations such as those referring to the activation of dormant origins during replicative stress [46],[47]. Thus, this model of origin regulation can help us to explain experimental observations that are not fully understood.

## Materials and Methods

### Pro-B Cell Cultures Preparation and Characterization

Short-term bone marrow pro-B cell cultures were prepared as previously described [48]. Briefly, bone marrow cells were isolated from an 8-wk-old *Rag2* ^−*/*−^ mouse (129/Sv) and plated at 10^6^ cells/ml in RPMI supplemented with 5% heat-inactivated fetal calf serum (FCS; Invitrogen), 2 mM L-glutamine, 1× penicillin-streptomycin, 50 μM 2-mercaptoethanol, and 10 ng/ml IL-7 (R&D). Cells were kept in culture for 7 d before labeling the replicating DNA with halogenated nucleotides. FACS analysis performed before DNA labeling indicated that more than 99% of the cells in culture were CD19^+^ (corresponding to committed pro-B cells). In contrast, pro-B cells from *Pax5* ^−*/*−^ and *Pax5* ^−*/*−^ *Rag2* ^−*/*−^ mice can be grown ex vivo for extended periods of time [49]. Long-term uncommitted pro-B cell cultures were prepared from the bone marrows of 1-wk-old *Pax5* ^−*/*−^ *Rag2* ^−*/*−^ (129/Sv) and *Pax5* ^−*/*−^ (129/Sv-C57BL/6) mice, as previously described [20]. These cells were expanded ex vivo on irradiated feeder cells in IMDM medium supplemented with 2% heat inactivated FCS, 0.15% primatone, 2 mM L-glutamine, 1× penicillin-streptomycin, 50 μM 2-mercaptoethanol, and 1 ng/ml IL-7. The mouse stromal cell line ST2 was used to prepare the feeder layers for pro-B cell cultures. These cells were grown in IMDM medium supplemented with 10% heat-inactivated FCS, 2 mM L-glutamine, 1× penicillin-streptomycin until 60% confluent, then irradiated (11 Gy) and used as feeders. All pro-B cell cultures were maintained as polyclonal populations. However, a clonal population was also derived from the bone marrow of a *Pax5* ^−*/*−^ mouse (129/Sv-C57BL/6) and used for some of the experiments presented in this study.

The location of Pax5 binding sites in committed pro-B cells, as well as their DNA sequence, chromatin features, and transcriptional activity, is presented for the entire *Igh* locus in a previous publication [28]. Briefly, within the 1.4 Mb region considered in the current study (from strain 129/Sv), the DNA sequences for six of these sites are present at 11 different locations (marked from 1 to 11 in Figure 4E). The sites marked from 1 to 4 have unique DNA sequences that align to the reference mouse genome (strain C57BL/6) at the following positions: assembly mm8_ chr12:<113676000113676290; chr12:113768900-113769250; chr12:113895270-113895500; and chr12:113972530-113972740. In contrast, sites 5 and 11 have the same DNA sequence and align to the reference strain at position mm8_chr12: 114630500-114630900, while sites 6–10 align at position mm8_chr12:114541600-114542000. With the exception of site number 4, all other sites display active chromatin marks in committed pro-B cells (H3K9ac and H3K4me2 [28]). However, these marks depend on Pax5 binding only at sites 1 and 2. Hence, there is no simple correlation between the firing rate of origins and the presence of active chromatin marks. Moreover, unlike Pax5 binding sites located in the distal portion of the *Igh* locus, none of these sites is associated with PAIR elements and Pax5-dependent antisense transcription [28].

A portion of the uncommitted pro-B cell culture was transduced with the retroviral vector *Pax5ER*-IRES-*GFP* that we previously used to reconstitute Pax5 expression in uncommitted pro-B cells [20],[30],[31],[50]. In these experiments, GFP expression allowed us to identify the cells successfully transduced with the retroviral vector. The populations of GFP^+^ cells were isolated by FACS in sterile conditions using a FACS MoFlo apparatus (sorting buffer: 1× PBS, 4% FBS, 1× penicillin-streptomycin). We have previously shown that the fusion protein Pax5ER can respond within minutes of the addition of 4-OHT (1 μM), resulting in the efficient expression of target genes (usually within a few hours from induction [30]). Pax5ER expression was monitored by immunoblot using antibodies raised against the amino terminus of Pax5 (Santa Cruz), or against the carboxy terminus of the estrogen receptor alpha (Santa Cruz), to determine the total level of expression in 3×10^5^ cells, using the expression of beta-actin as a reference (Santa Cruz). The induction of Pax5ER was monitored by FACS using anti-mouse-CD19 antibody (Pharmingen). Following 4-OHT induction, CD19^+^ and CD19^−^ pro-B cells were isolated using an AutoMACS cell separator and anti-CD19 conjugated magnetic beads (Miltenyi), according to the manufacturer's procedure. Cell-cycle analysis before and after 4-OHT induction was performed by FACS following propidium-iodide staining of the cells (50 mg/L) in hypotonic buffer (0.1% sodium citrate). The lack of DNA-damage checkpoint activation was determined on total cell lysates from 3×10^5^ cells in the presence of a protease and phosphatase-inhibitors cocktail (Thermo Scientific), using antibodies raised against p53 (Cell Signaling), phospho-p53, Ser15 (Cell Signaling), phospho-histone H2A.X, Ser139 (Cell Signaling), phospho-Chk1, Ser345 (Cell Signaling), phospho-Rad17, Ser645 (Cell Signaling), and phospho-ATM, Ser1981 (Santa Cruz).

### Double-Labeling of Replicating DNA, DNA Purification, and SMARD

A detailed procedure for SMARD is presented in previous publications [17],[21],[22]. Briefly, cells in the exponential phase of growth were sequentially labeled with IdU and CldU (25 μM each) for periods of time between 3 and 4 h at a density between 3×10^5^ and 8×10^5^ cells/ml. The duration of each of the labeling periods was chosen to be longer than the replication time of the fragments analyzed but short enough to prevent multiple rounds of replication according to the principles described in previous publications [17],[21]. Total genomic DNA was prepared in agarose plugs (about 10^6^ cells/plug) and digested with either *Pac*I, *Swa*I, or *Pme*I (40 units/plug). Restriction fragments were purified by pulsed-field gel electrophoresis, recovered by agarase digestion, and stretched on silanized microscope slides by capillary action. During SMARD, the restriction fragments of interest were detected with the following probes: *Swa*I #2 was detected with several PCR products spanning the positions 14,606–25,310 and 35,667–46,206 of the *Igh* clone BAC199M11 (GeneBank: AF450245), together with a plasmid clone containing the switch region γ1 (pγ1/EH10; [51],[52]); *Pac*I #3 was detected with the plasmids UUGC2M0237A15 and UUGC2M0215F07 (GeneBank: AZ966978, AZ966626/AZ951255, AZ950883), and with PCR products spanning, respectively, positions 9530909–9543550, 9560850–9571463, and 9644195–9652184 in the mouse chromosome 12 contig (GeneBank: NT_039553); *Pme*I #4 was detected with PCR products spanning, respectively, positions 1204509–1206646, 1206954–1208600, 1209395–1211337, and 1212606–1213479 in GeneBank: AJ851868; and *Pme*I #5 was detected with the plasmids UUGC1M0532J03, UUGC1M0259G14, UUGC2M0265N20, UUGC1M0166E11, and UUGC2M0258M14 (spanning positions 211454–219261, 413828–421730, 591439–598359, 631282–640007, and 647267-663409 in GeneBank: AJ851868). The same probes were used for the detection of both the 129/Sv and the C57BL/6 alleles. Microscopy image alignment to the maps of the restriction fragments and data analysis were performed as previously described [22]. Only molecules unbroken, fully-substituted with halogenated nucleotides, and sufficiency stretched to be aligned were considered.

### Numerical Simulation of the SMARD Data

In order to determine how origin firing is regulated in mammalian cells, we developed a new mathematical formalism and a simulation procedure which allow us to rigorously analyze the experimental results obtained by SMARD. The numerical simulations used in this study allow us to reproduce SMARD data sets from any given known replication scenario (i.e., a defined firing rate and fork speed), making it possible for us to study the statistics of the experimental data sets. As reported in the Results section, we performed numerical simulations of all experiments presented in this study. The simulation procedure is described in detail in reference [18] but can be summarized as follows:

1. A replication cycle is simulated using a Monte-Carlo procedure. Replication forks are stochastically created based on firing rates similar to the ones presented in Figure 2E. Then the DNA is bi-directionally replicated from each origin by forks moving at a constant speed until all forks coalesce.
2. Once the full replication cycle is simulated, the labeling procedure is also mimicked by randomly defining the time when incorporation of halogenated nucleotides starts relative to the starting time of the replication cycle.
3. A labeled fragment is generated from the simulated replication cycle and the labeling timeline. The simulations give us the positions of the forks as a function of time. The labeled fragments are created from the fork positions at the times when nucleotide types are switched.
4. Steps 1 to 3 are repeated many times (typically 5,000 times), and the recorded simulated fragments are analyzed just as the real molecules were. In particular, when the number of analyzed fragments equals that of the actual experiments, we can use the simulation-to-simulation variations of measured quantities to estimate errors of those quantities. This leads to the error bars shown in various figures, such as Figure 2B–C (fork densities), Figure 2D (IdU content), and Figure S2B (replication time).

### Computer Modeling of the SMARD Data

In principle, numerical simulations could also be used to determine the most likely set of parameters responsible for generating specific experimental data sets obtained by SMARD. However, the need to explore a large number of variables makes this fitting procedure impractical and time consuming when the replication scenario is not pre-determined. Therefore, we also developed an analytical formalism that allows us to rapidly model the results obtained by SMARD [18]. The formalism leads to rate equations that describe changes in the number of replication forks as a function of both the location along the genome (*x*) and the time elapsed since the beginning of S phase (*t*). More specifically, we define a set of three coupled partial differential equations that describe the space-time evolution of the replication fraction, *f*_(*x*, *t*)_, and the two densities of forks, *ρ*±(*x*, *t*), moving rightward (+) and leftward (–). Equation 1 gives the rate of change of the probability that a specific location *x* along the genome is replicated at time *t*,:
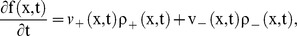
where *v*±(*x*, *t*) is the speed of the replication fork at the position *x* at time *t*. This first equation states that the rate of replication of a specific location is proportional to the number of forks at the same location times their respective replication time. The rate of change of the fork densities (i.e., the number of forks moving in a given direction per kb) is given by Equation 2:
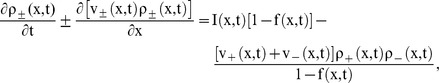
where *I*(*x*, *t*) is the firing rate that corresponds to the number of initiation events per time per unreplicated DNA length. The two terms on the right-hand side of the latter equation represent initiation and termination events, respectively, while the terms on the left-hand side express the space-time-propagation of the replication forks. This second equation defines the rate of change of the probability of observing a replication fork at a specific location and time in the form of a classic transport equation. If both the firing rate and the fork speed space-time functions are known, these equations can be numerically integrated to obtain *f*(*x*, *t*) and *ρ*±(*x*, *t*). Once our set of equations is solved, the results can be converted to profiles of IdU content and fork distributions, as they are observed in the SMARD experiment (for details, see reference [18]). The solution can also be used to calculate the average number of initiation events occurring within a specific section of the locus, per allele, during each S phase as:
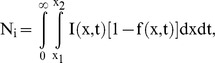
where *x_1_* and *x_2_* are the boundaries of the region of interest (Equation 3). This calculation approach provides us with a faster and more precise way to describe the average replication kinetics for a specific genomic region, and its results were validated using simulations of known replication scenarios, as described in the previous section. In addition, simulations are still needed in order to study the statistical fluctuations of *f*(*x*, *t*) and *ρ*±(*x*, *t*).

### Fitting Experimental Data

This calculation technique was then used to fit the experimental data sets to parameterized functional forms for *I*(*x*, *t*) and *v*±(*x*, *t*). This means that starting with a series of parameters defining both the firing and the velocity profiles, we used our calculation technique to produce the corresponding SMARD-like results. The parameters were then iteratively adjusted in order to obtain the best fit between the calculated values and the experimental measurements. For the fits performed in this study, we assumed homogeneous fork speeds throughout both space and time (i.e., *v*±(*x*, *t*)  =  *v* for left- and right-moving forks). We also assumed that the firing rate is constant throughout time but spatially inhomogeneous, *I*(*x*, *t*)  =  *I*(*x*). A larger firing rate implies a shorter average replication time and vice versa. Time-dependent firing rates were considered but were not needed to obtain a satisfactory representation of the data. In other words, at the hundred-kilobase-to-megabase scale considered in this study, the replication timing is dominated by spatial variations in the firing rate. Our calculation technique also provided us the distribution of times required to duplicate a given section of the genome.

The SMARD data used in the fit are shown as empty symbols in Figures 2B–D and S2B. For each experiment, we performed a global fit of the two replication fork distributions (solid lines in Figure 2B–C) of the IdU content (solid lines in Figure 2D), and of the replication times for all restriction fragments (filled symbols in Figure S2B). Error bars on the experimental data were obtained from numerical simulations where each simulated experiment uses the best-fit parameters to collect the same number of labeled DNA fragments analyzed in the real experiment. Figure S2D shows the free parameters obtained from the fits, with the firing rate of origins *I*(*x*) also graphically illustrated in Figure 2E. For the purpose of the calculations, the firing rate was considered to be composed by a constant background of initiation events (one parameter), to which additional events are added where initiations are more likely to occur (active origins clusters). For simplicity, these initiation clusters were assumed to have the shape of a rounded box defined by three parameters (position and width of the cluster, and the firing rate within the cluster). While this is likely to represent an oversimplification of the conditions existing in vivo, we found that more complex shapes were not required to obtain meaningful representations of the experimental data sets. In contrast, Gaussian curves were tested but resulted in fits that do not accurately reproduce the observed number of initiation events and their location. Hence, curves where the firing rate changes sharply along the genome provide a better representation of the experimental data sets than curves where the firing rate changes gradually. In Figure 2E, two additional parameters show the level of origin firing outside the genomic region investigated. The value of these parameters may be seen as an effective constant firing rate profile outside the studied region. Finally, one parameter was used to fit the velocity of fork propagation (meaning that the speed of replication forks was assumed to be constant throughout the region analyzed). Among the other results of the fit, Figure S2F shows the probability for each section of the genome to be replicated by forks that originated within the modeled region, while Figure S2G shows the probability of being replicated by replication forks moving leftward or rightward. Finally, Figure 2F shows the average replication kinetics of the modeled genomic region. The color value on this space-time graph indicates the probability for each genomic position to replicate at a specific time in S phase. The transition from unreplicated (orange) to replicated (blue) occurs at a different speed in a different portion of the genome. Three contour lines are shown to illustrate which regions replicate faster than others (showing when 10%, 50%, and 90% of cells have replicated the corresponding portion of the genome).

The images collected by SMARD represent all stages of DNA replication. As a result, the number of initiation and termination events scored on individual DNA molecules (Table S1, columns g and h) is lower than the actual number of events taking place during every replication cycle. For example, initiation events can be easily scored when the size of the IdU patch is small, but no longer so at more advanced stages of replication (when one of the outward-moving forks runs off the fragment or coalesces with another fork). In contrast, our calculation method determines the actual number of initiation events and fork collisions occurring, on average, every replication cycle. Hence, in order to compare the outcomes of these two procedures, we performed additional simulations of the SMARD experiments to determine how many of initiations/collisions would be observed experimentally from the results of each fit. Figure S2C shows that the number of events obtained from the fit is comparable to the number observed experimentally.

## Supporting Information

Figure S1 Single molecule analysis of replicated DNA. DNA labeling scheme used for SMARD. Within a population of exponentially growing cells, DNA synthesis is limited to cells transiting through the S phase. If a specific portion of the cellular genome is considered (e.g., a restriction fragment, gray bars), the distribution of replication forks within this region will be representative of all stages of DNA replication (steady state distribution). Following a first labeling period of 3 h with IdU (central panel), each cell will be at a different position in the cell cycle and the halogenated nucleotide will be distributed among the DNA molecules as indicated by the red color. Only the DNA molecules that started and completed their replication during the labeling period will be fully substituted with IdU, while the others will be either partially substituted or not labeled. Following a second labeling period of 3 h with CldU (right panel), halogenated nucleotides will be distributed among the DNA molecules as indicated by the red and green colors (IdU and CldU, respectively). During SMARD, only the molecules fully substituted with one or both nucleotide analogs are considered. Within the population of double-labeled DNA molecules, the distribution of IdU-CldU transitions corresponds to the distribution of replication forks at the time of the label switch.
(TIF)

Figure S2 Replication of the *Igh* locus in *Pax5* ^−*/*−^ *Rag2* ^−*/*−^ uncommitted pro-B cells (129/Sv). (A) Image of a double-labeled DNA molecule containing two initiation events and two externally generated replication forks. (B) Graphical representation of the replication times (*Tr*) for each restriction fragment obtained experimentally by SMARD (gray; see Table S1 for details) and from the fit (red). Error bars were calculated as described in Materials and Methods. (C) Numbers and percentages of initiation events and fork collisions detected in the population of double-labeled DNA molecules by SMARD, or calculated from the fit. (D) Miscellaneous results from the fit including the most likely size and position of the active origin clusters. (E) Schematics of the four restriction fragments analyzed in this experiment (shown to scale). Black ovals indicate the position of the midpoints for the initiation events detected in these experiments (where the corresponding origins are more likely to be located). Multiple ovals overlapping the same portion of the genome are a hallmark of origin mapping saturation (it indicates that the same origin has been mapped on different DNA molecules). The rarity of these overlaps indicates that origin density is higher than depicted. (F) Probability for various portions of the locus to be replicated by origins located within the genomic region under investigation (yellow), or near the distal-V_H_ genes (red), or downstream of the *Igh* locus (blue), as calculated from the fit. (G) Probability for various portions of the locus of being replicated by forks moving rightward (red) or leftward (blue), as calculated from the fit. Note that the probability changes in relation to the firing rate of origins (gray dotted line).
(TIF)

Figure S3 Replication of the *Igh* locus in a D_H_-J_H_ rearranged clonal population of *Pax5* ^−*/*−^ uncommitted pro-B cells (129/Sv-C57BL/6). The results for each allele are shown separately. (A–F) Summary of some of the results obtained by SMARD and from the fitting procedure (as described for Figure S2).
(TIF)

Figure S4 Replication of the *Igh* locus in *Rag2* ^−*/*−^ committed pro-B cells (129/Sv). (A–F) Summary of some of the results obtained by SMARD and from the fitting procedure (as described for Figure S2). For three of the restriction fragments analyzed in these cells, the number of double-labeled DNA molecules displayed the occurrence of more than one initiation event is significantly higher than in uncommitted pro-B cells (9%, 14%, and 12%, respectively, for *SwaI* #2, *PmeI* #4, and *PmeI* #5). This is consistent with an increase in the average number of origins firing within the locus during each replication cycle (see main text).
(TIF)

Figure S5 Reconstitution of Pax5 expression in *Pax5* ^−*/*−^ uncommitted pro-B cells (129/Sv-C57BL/6). (A) Level of expression of Pax5ER determined using a monoclonal antibody that recognizes the amino-terminal portion of Pax5. Before 4-OHT induction, the level of Pax5ER in KO-Pax5ER pro-B cells is approximately 20% of the level of *wt* Pax5 present in the pro-B cell line 6312. (B) Following 4-OHT induction, the expression of Pax5ER increases several times, to reach levels comparable to *wt* Pax5. (C) Kinetics of expression of the surface marker CD19 following 4-OHT induction. A low percentage of CD19^+^ cells are present before the addition of 4-OHT, but this value increases more than 20 times after the induction of Pax5ER. A small portion of the KO-Pax5ER pro-B cells remains CD19^−^ even after prolonged 4-OHT induction. (D–I) Summary of some of the results obtained by SMARD and from the fitting procedure (as described for Figure S2). (L) Cell cycle profiles of KO-Pax5ER pro-B cells before (0 h) and after 4-OHT induction (3 h and 24 h). These cells continue to cycle even after 4-OHT treatments longer than 1 wk (not shown). (M) Absence of markers of DNA damage and checkpoints'activation following 4-OHT induction. Immunoblots from two different reconstitution experiments (*Pax5* ^−*/*−^ and *Pax5* ^−*/*−^ *Rag2* ^−*/*−^) are shown before (−) and after (+) a 3 h treatment with 1 μM 4-OHT. Similar results were obtained using induction times between 1 and 24 h, and with antibodies against phospho-ATM Ser1981, and phospho-Rad17 Ser645 (not shown). The positive controls for each immunoblot (E) were prepared by treating KO-Pax5ER pro-B cells with 25 μM etoposide for 2 h.
(TIF)

Figure S6 Kinetic of the changes in origin activity induced by 4-OHT (and by 4-OHT withdrawal) within the C_H_-3′RR region in KO-Pax5ER pro-B cells. (A–F) Summary of some of the results obtained by SMARD and from the fitting procedure (as described for Figure S2). All experimental data sets obtained for the 129/Sv allele were fitted simultaneously as described in Materials and Methods. Panel C also shows the positions of the initiation and termination events detected by SMARD, with numerals indicating the normalized frequency of the events as described for Figure 2.
(TIF)

Figure S7 Labeling of DNA molecules from fragment *SwaI#2* during SMARD. Results presents in Table 1 indicate that *SwaI#2* requires approximately 60′to complete its replication. At the top of the picture, this window of time is presented as a gray double-headed arrow. In an asynchronous population of cells, this window of time will be variably distributed in relation to the labeling periods with IdU and CldU (see double-headed arrows labeled from1 to 36). This will result in molecules that are variably substituted with the nucleotide (unlabelled if the molecule started replicating before time *a*, partially IdU-labeled if the molecule started replicating between time *a* and *b*, fully IdU-labeled between time *b* and *c*, double-labeled between time *c* and *d*, and so on). For each cell, the window always encompasses a portion of S phase. From Figures, 2F, 4F, and 5H we know that the replication of fragment *SwaI#2* is complete in more than 90% of cells by 140′ minutes into S phase. Since the fragment takes approximately 60′ to replicate, we conclude that the replication of each individual molecule starts between 0′ and 80′ (140 – 60  =  80) into S phase (meaning that the G1/S occurs from 0′ to 80′ before each fragment starts replicating). Black double-headed arrows indicate the possible location of the G1/S transition for the cells that produce the population of double labeled molecules. We can see that when 4-OHT is provided 1 h before the label switch a few cells will have already passed the G1/S transition (e.g., #16), while many others will have a similar probability of being at the end of G1 or in early S (e.g., #19–22).
(TIF)

Table S1 Quantitative analysis of the SMARD data. Column a, genetic background, cell type and treatment, restriction fragment name, and mouse strain(s) of origin for the *Igh* loci analyzed in each experiment. The name of the mouse strain repeated twice indicates that the cells used for the experiment were homozygous and that the two *Igh* alleles were analyzed together. In all other cases, the *Igh* alleles were analyzed independently and only the corresponding mouse strain is indicated. ΔD_H_-J_H_ indicates that the *Igh* allele was D_H_-J_H_ rearranged. Column b, duration of the first and second labeling periods with IdU and CldU (respectively, Tp1 and Tp2). SMARD requires that the duration of each labeling period is longer than the average replication time of the restriction fragments under investigation [17],[22]. Column c, number of *Igh* molecules surveyed by fluorescence microscopy. This number includes all the molecules detected during each experiment based on the presence of the hybridization signals and regardless of the level of substitution with halogenated nucleotides (e.g., fully, partially, or not substituted). In some of the experiments only the molecules fully substituted with halogenated nucleotides were counted; therefore, this value was not determined (n.d.). Columns d and e, respectively, percentage and number of surveyed molecules containing one or both halogenated nucleotides. Only some of these molecules fulfilled the criteria required by SMARD and were included in column f (see below). Column f, N_R_, N_G_, and N_RG_ indicate the number of imaged molecules substituted with, respectively, IdU (detected in red), CldU (detected in green), and both halogenated nucleotides (double-labeled, both red and green). These numbers include only the molecules suitable for measurement (fully substituted, unbroken, sufficiently stretched to be unequivocally aligned to the map of the region, and not overlapping with other molecules). Column g, number of initiation events normalized for 100 double-labeled DNA molecules (in red) and percentage of double-labeled DNA molecules containing initiation events (in black). These values may differ for some of the restriction fragments because a fraction of the DNA molecules can contain more than one initiation event. Column h, number of colliding forks (termination events) normalized for 100 double-labeled DNA molecules (in red) and percentage of double-labeled DNA molecules containing colliding forks (in black). Column i, average time required to replicate the genomic region encompassed by the restriction fragment. This value (Tr) was calculated using the following equation: Tr  =  Tp2 × NRG/(NG+NRG). A detailed description of the procedure is provided in previous publications [17],[22]. Column l, average number of replication forks within the population of double-labeled DNA molecules. Column m, average speed of replication forks within the restriction fragment. This value is obtained by dividing the size of the restriction fragment (from column n) by Tr (from column i) and by the average number of replication forks (from column l). The values indicated are expressed in kilobases per minute. Column n, size of the restriction fragment in kilobases.
(PDF)

## Abbreviations

4-OHT: 4-hydroxy-tamoxifen
CldU: 5′-chloro-2′-deoxyuridine
IAS: number of initiation events occurring within a specific section of the locus, per allele, per S phase
IdU: 5′-iodo-2′-deoxyuridine
pre-RC: pre-replication complex
SMARD: single molecule analysis of replicated DNA

## References

[1] Hiratani I, Ryba T, Itoh M, Rathjen J, Kulik M (2010). Genome-wide dynamics of replication timing revealed by in vitro models of mouse embryogenesis.. Genome Res.

[2] Norio P, Kosiyatrakul S, Yang Q, Guan Z, Brown N. M (2005). Progressive activation of DNA replication initiation in large domains of the immunoglobulin heavy chain locus during B cell development.. Mol Cell.

[3] Aladjem M. I, Rodewald L. W, Lin C. M, Bowman S, Cimbora D. M (2002). Replication initiation patterns in the beta-globin loci of totipotent and differentiated murine cells: evidence for multiple initiation regions.. Mol Cell Biol.

[4] Goren A, Tabib A, Hecht M, Cedar H (2008). DNA replication timing of the human beta-globin domain is controlled by histone modification at the origin.. Genes Dev.

[5] Kitsberg D, Selig S, Keshet I, Cedar H (1993). Replication structure of the human beta-globin gene domain.. Nature.

[6] Goldar A, Marsolier-Kergoat M. C, Hyrien O (2009). Universal temporal profile of replication origin activation in eukaryotes.. PLoS One.

[7] Rhind N, Yang S. C, Bechhoefer J (2009). Reconciling stochastic origin firing with defined replication timing.. Chromosome Res.

[8] Lygeros J, Koutroumpas K, Dimopoulos S, Legouras I, Kouretas P (2008). Stochastic hybrid modeling of DNA replication across a complete genome.. Proc Natl Acad Sci U S A.

[9] Blow J. J, Ge X. Q (2009). A model for DNA replication showing how dormant origins safeguard against replication fork failure.. EMBO Rep.

[10] Lucas I, Chevrier-Miller M, Sogo J. M, Hyrien O (2000). Mechanisms ensuring rapid and complete DNA replication despite random initiation in Xenopus early embryos.. J Mol Biol.

[11] Goldar A, Labit H, Marheineke K, Hyrien O (2008). A dynamic stochastic model for DNA replication initiation in early embryos.. PLoS One.

[12] Czajkowsky D. M, Liu J, Hamlin J. L, Shao Z (2008). DNA combing reveals intrinsic temporal disorder in the replication of yeast chromosome VI.. J Mol Biol.

[13] Guilbaud G, Rappailles A, Baker A, Chen C. L, Arneodo A (2012). Evidence for sequential and increasing activation of replication origins along replication timing gradients in the human genome.. PLoS Comput Biol.

[14] Rhind N (2006). DNA replication timing: random thoughts about origin firing.. Nat Cell Biol.

[15] Yang S. C, Rhind N, Bechhoefer J (2010). Modeling genome-wide replication kinetics reveals a mechanism for regulation of replication timing.. Mol Syst Biol.

[16] Retkute R, Nieduszynski C. A, de Moura A (2011). Dynamics of DNA replication in yeast.. Phys Rev Lett.

[17] Norio P, Schildkraut C. L (2001). Visualization of DNA replication on individual Epstein-Barr virus episomes.. Science.

[18] Gauthier M. G, Norio P, Bechhoefer J (2012). Modeling inhomogeneous DNA replication kinetics.. PLoS One.

[19] Hyrien O, Goldar A (2009). Mathematical modelling of eukaryotic DNA replication.. Chromosome Res.

[20] Nutt S. L, Urbanek P, Rolink A, Busslinger M (1997). Essential functions of Pax5 (BSAP) in pro-B cell development: difference between fetal and adult B lymphopoiesis and reduced V-to-DJ recombination at the IgH locus.. Genes Dev.

[21] Demczuk A, Norio P (2009). Determining the replication dynamics of specific gene loci by single-molecule analysis of replicated DNA.. Methods Mol Biol.

[22] Norio P, Schildkraut C. L (2004). Plasticity of DNA replication initiation in Epstein-Barr virus episomes.. PLoS Biol.

[23] Vaughn J. P, Dijkwel P. A, Hamlin J. L (1990). Replication initiates in a broad zone in the amplified CHO dihydrofolate reductase domain.. Cell.

[24] Little R. D, Platt T. H, Schildkraut C. L (1993). Initiation and termination of DNA replication in human rRNA genes.. Mol Cell Biol.

[25] Berezney R, Dubey D. D, Huberman J. A (2000). Heterogeneity of eukaryotic replicons, replicon clusters, and replication foci.. Chromosoma.

[26] Kalejta R. F, Li X, Mesner L. D, Dijkwel P. A, Lin H. B (1998). Distal sequences, but not ori-beta/OBR-1, are essential for initiation of DNA replication in the Chinese hamster DHFR origin.. Mol Cell.

[27] Mesner L. D, Li X, Dijkwel P. A, Hamlin J. L (2003). The dihydrofolate reductase origin of replication does not contain any nonredundant genetic elements required for origin activity.. Mol Cell Biol.

[28] Ebert A, McManus S, Tagoh H, Medvedovic J, Salvagiotto G (2011). The distal V(H) gene cluster of the Igh locus contains distinct regulatory elements with Pax5 transcription factor-dependent activity in pro-B cells.. Immunity.

[29] Holmes M. L, Carotta S, Corcoran L. M, Nutt S. L (2006). Repression of Flt3 by Pax5 is crucial for B-cell lineage commitment.. Genes Dev.

[30] Nutt S. L, Morrison A. M, Dorfler P, Rolink A, Busslinger M (1998). Identification of BSAP (Pax-5) target genes in early B-cell development by loss- and gain-of-function experiments.. EMBO J.

[31] Schebesta A, McManus S, Salvagiotto G, Delogu A, Busslinger G. A (2007). Transcription factor Pax5 activates the chromatin of key genes involved in B cell signaling, adhesion, migration, and immune function.. Immunity.

[32] Giambra V, Volpi S, Emelyanov A. V, Pflugh D, Bothwell A. L (2008). Pax5 and linker histone H1 coordinate DNA methylation and histone modifications in the 3′ regulatory region of the immunoglobulin heavy chain locus.. Mol Cell Biol.

[33] Cobaleda C, Schebesta A, Delogu A, Busslinger M (2007). Pax5: the guardian of B cell identity and function.. Nat Immunol.

[34] Remus D, Diffley J. F (2009). Eukaryotic DNA replication control: lock and load, then fire.. Curr Opin Cell Biol.

[35] Desprat R, Thierry-Mieg D, Lailler N, Lajugie J, Schildkraut C (2009). Predictable dynamic program of timing of DNA replication in human cells.. Genome Res.

[36] McCune H. J, Danielson L. S, Alvino G. M, Collingwood D, Delrow J. J (2008). The temporal program of chromosome replication: genomewide replication in clb5{Delta} Saccharomyces cerevisiae.. Genetics.

[37] Knott S. R, Peace J. M, Ostrow A. Z, Gan Y, Rex A. E (2012). Forkhead transcription factors establish origin timing and long-range clustering in S. cerevisiae.. Cell.

[38] Hassan-Zadeh V, Chilaka S, Cadoret J. C, Ma M. K, Boggetto N (2012). USF binding sequences from the HS4 insulator element impose early replication timing on a vertebrate replicator.. PLoS Biol.

[39] Mantiero D, Mackenzie A, Donaldson A, Zegerman P Limiting replication initiation factors execute the temporal programme of origin firing in budding yeast.. EMBO J.

[40] Tanaka S, Nakato R, Katou Y, Shirahige K, Araki H Origin association of Sld3, Sld7, and Cdc45 proteins is a key step for determination of origin-firing timing.. Curr Biol.

[41] Guo C, Gerasimova T, Hao H, Ivanova I, Chakraborty T (2011). Two forms of loops generate the chromatin conformation of the immunoglobulin heavy-chain gene locus.. Cell.

[42] Sayegh C. E, Jhunjhunwala S, Riblet R, Murre C (2005). Visualization of looping involving the immunoglobulin heavy-chain locus in developing B cells.. Genes Dev.

[43] Fuxa M, Skok J, Souabni A, Salvagiotto G, Roldan E (2004). Pax5 induces V-to-DJ rearrangements and locus contraction of the immunoglobulin heavy-chain gene.. Genes Dev.

[44] Schwab K. R, Patel S. R, Dressler G. R (2011). The role of PTIP in class switch recombination and long range chromatin interactions at the immunoglobulin heavy chain locus.. Mol Cell Biol.

[45] Wong P. G, Winter S. L, Zaika E, Cao T. V, Oguz U (2011). Cdc45 limits replicon usage from a low density of preRCs in mammalian cells.. PLoS One.

[46] Ge X. Q, Jackson D. A, Blow J. J (2007). Dormant origins licensed by excess Mcm2-7 are required for human cells to survive replicative stress.. Genes Dev.

[47] Ge X. Q, Blow J. J (2010). Chk1 inhibits replication factory activation but allows dormant origin firing in existing factories.. J Cell Biol.

[48] Johnson K, Angelin-Duclos C, Park S, Calame K. L (2003). Changes in histone acetylation are associated with differences in accessibility of V(H) gene segments to V-DJ recombination during B-cell ontogeny and development.. Mol Cell Biol.

[49] Urbanek P, Wang Z. Q, Fetka I, Wagner E. F, Busslinger M (1994). Complete block of early B cell differentiation and altered patterning of the posterior midbrain in mice lacking Pax5/BSAP.. Cell.

[50] Holmes M. L, Pridans C, Nutt S. L (2008). The regulation of the B-cell gene expression programme by Pax5.. Immunol Cell Biol.

[51] Mowatt M, Dery C, Dunnick W (1985). Unique sequences are interspersed among tandemly repeated elements in the murine gamma 1 switch segment.. Nucleic Acids Res.

[52] Mowatt M. R, Dunnick W. A (1986). DNA sequence of the murine gamma 1 switch segment reveals novel structural elements.. J Immunol.

