# Supplementary figures and images for: Regulation of DNA Replication within the Immunoglobulin Heavy-Chain Locus During B Cell Commitment
Source: PLoS Biol. 2012 Jul 10;10(7):e1001360. doi: 10.1371/journal.pbio.1001360 (PMC3393677; doi:10.1371/journal.pbio.1001360)

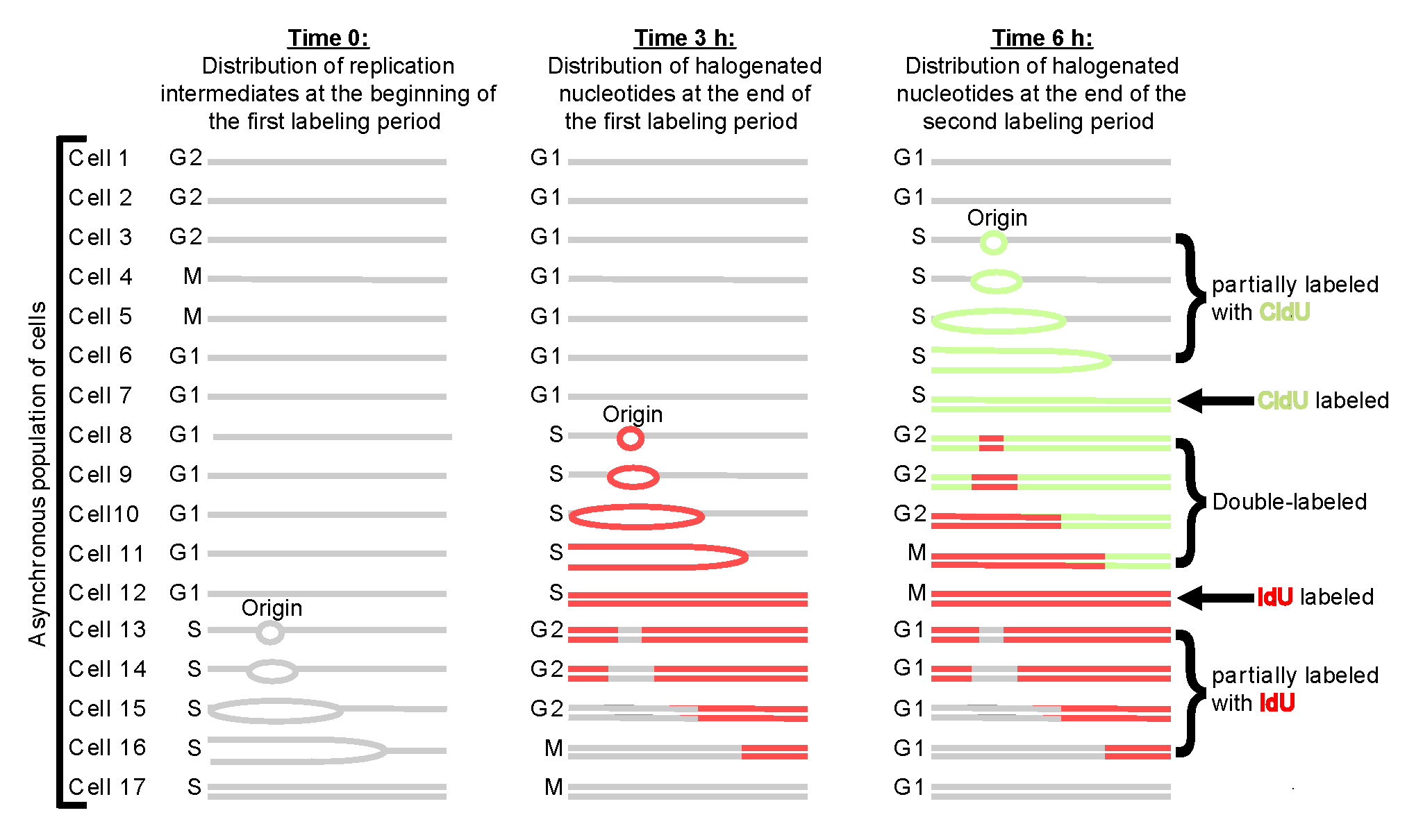

Supplement: Figure S1 — Single molecule analysis of replicated DNA. DNA labeling scheme used for SMARD. Within a population of exponentially growing cells, DNA synthesis is limited to cells transiting through the S phase. If a specific portion of the cellular genome is considered (e.g., a restriction fragment, gray bars), the distribution of replication forks within this region will be representative of all stages of DNA replication (steady state distribution). Following a first labeling period of 3 h with IdU (central panel), each cell will be at a different position in the cell cycle and the halogenated nucleotide will be distributed among the DNA molecules as indicated by the red color. Only the DNA molecules that started and completed their replication during the labeling period will be fully substituted with IdU, while the others will be either partially substituted or not labeled. Following a second labeling period of 3 h with CldU (right panel), halogenated nucleotides will be distributed among the DNA molecules as indicated by the red and green colors (IdU and CldU, respectively). During SMARD, only the molecules fully substituted with one or both nucleotide analogs are considered. Within the population of double-labeled DNA molecules, the distribution of IdU-CldU transitions corresponds to the distribution of replication forks at the time of the label switch. (TIF) [file pbio.1001360.s001.tif]

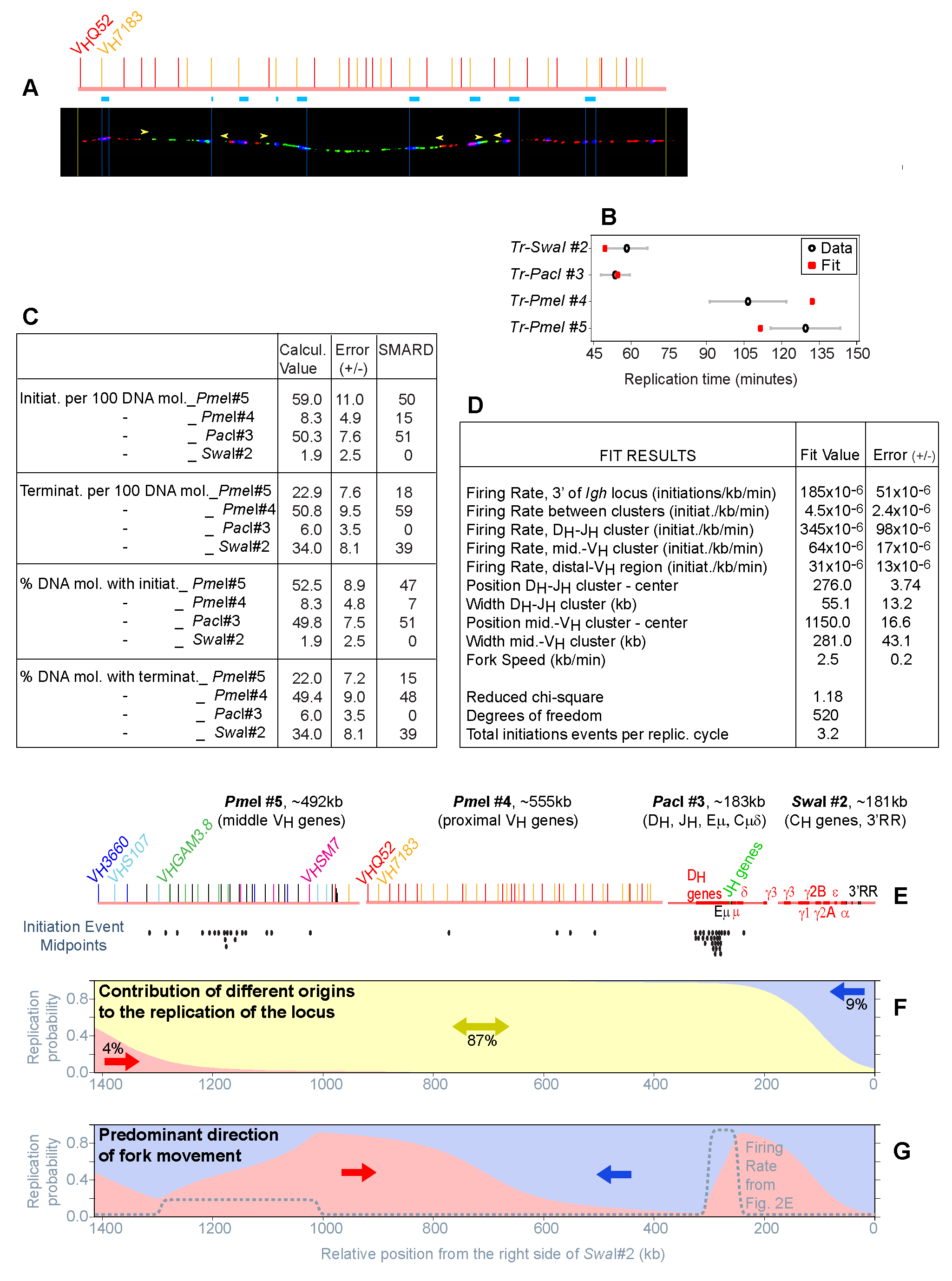

Supplement: Figure S2 — Replication of the Igh locus in Pax5 −/− Rag2 −/− uncommitted pro-B cells (129/Sv). (A) Image of a double-labeled DNA molecule containing two initiation events and two externally generated replication forks. (B) Graphical representation of the replication times (Tr) for each restriction fragment obtained experimentally by SMARD (gray; see Table S1 for details) and from the fit (red). Error bars were calculated as described in Materials and Methods. (C) Numbers and percentages of initiation events and fork collisions detected in the population of double-labeled DNA molecules by SMARD, or calculated from the fit. (D) Miscellaneous results from the fit including the most likely size and position of the active origin clusters. (E) Schematics of the four restriction fragments analyzed in this experiment (shown to scale). Black ovals indicate the position of the midpoints for the initiation events detected in these experiments (where the corresponding origins are more likely to be located). Multiple ovals overlapping the same portion of the genome are a hallmark of origin mapping saturation (it indicates that the same origin has been mapped on different DNA molecules). The rarity of these overlaps indicates that origin density is higher than depicted. (F) Probability for various portions of the locus to be replicated by origins located within the genomic region under investigation (yellow), or near the distal-VH genes (red), or downstream of the Igh locus (blue), as calculated from the fit. (G) Probability for various portions of the locus of being replicated by forks moving rightward (red) or leftward (blue), as calculated from the fit. Note that the probability changes in relation to the firing rate of origins (gray dotted line). (TIF) [file pbio.1001360.s002.tif]

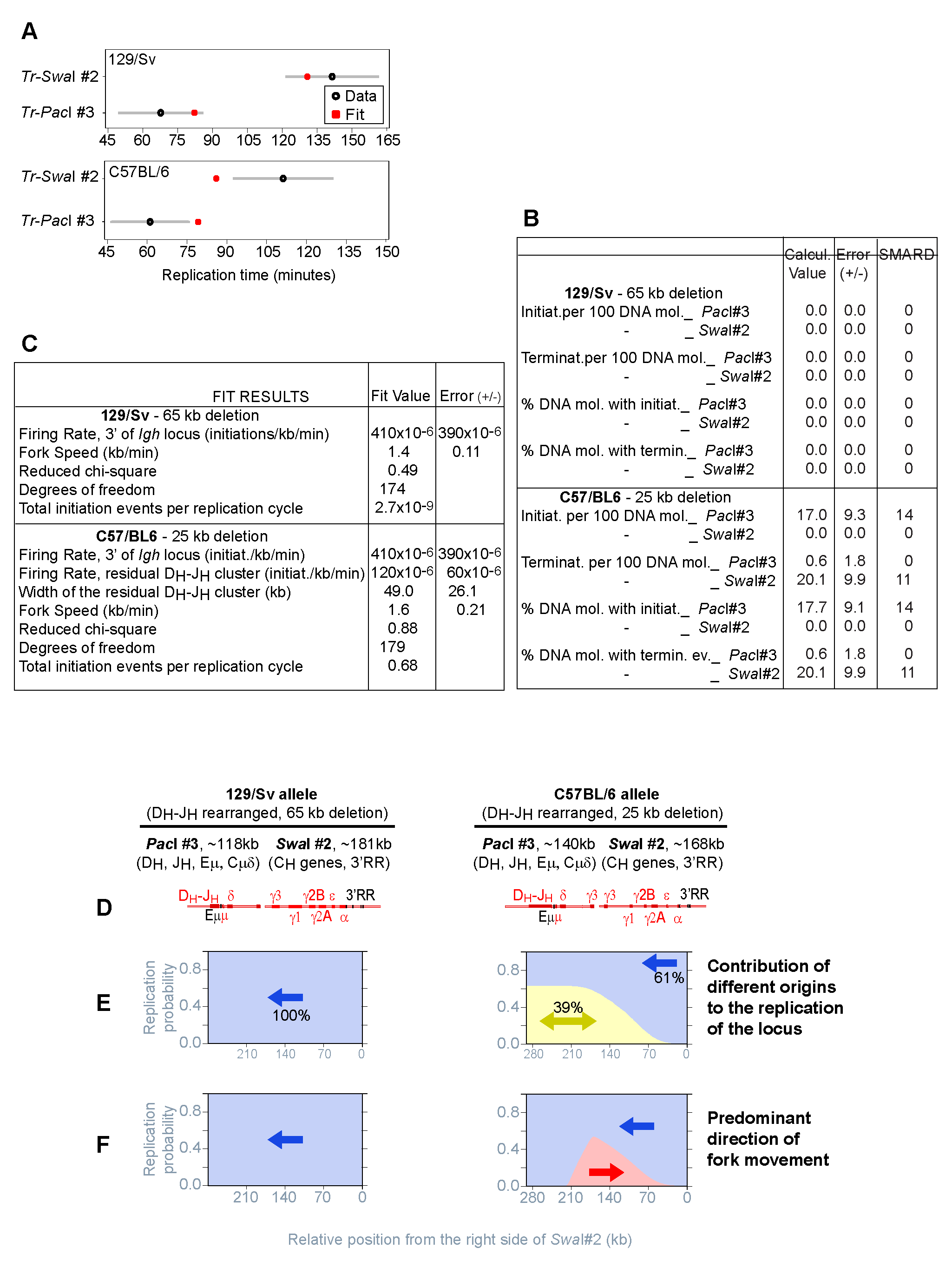

Supplement: Figure S3 — Replication of the Igh locus in a DH-JH rearranged clonal population of Pax5 −/− uncommitted pro-B cells (129/Sv-C57BL/6). The results for each allele are shown separately. (A–F) Summary of some of the results obtained by SMARD and from the fitting procedure (as described for Figure S2). (TIF) [file pbio.1001360.s003.tif]

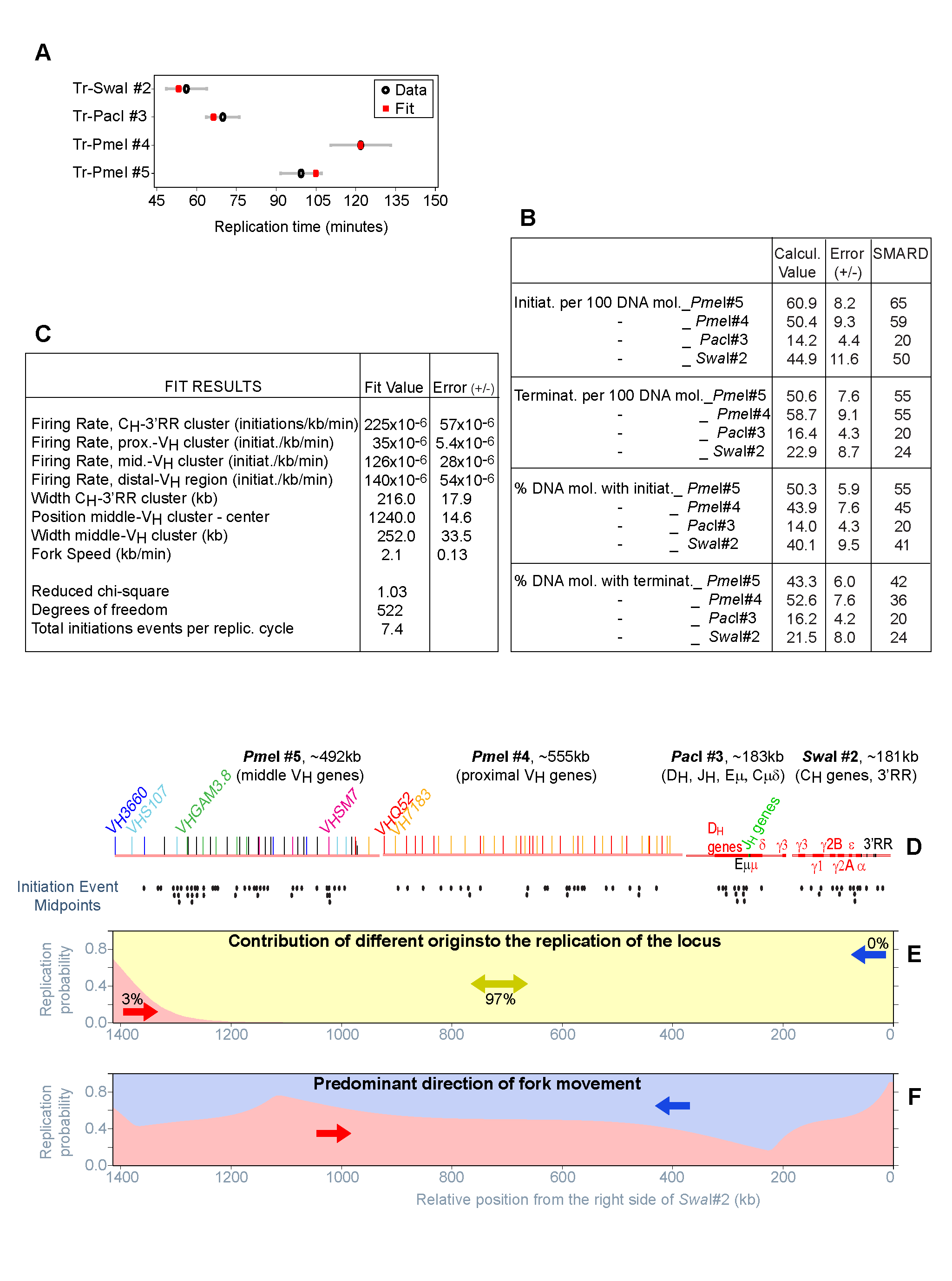

Supplement: Figure S4 — Replication of the Igh locus in Rag2 −/− committed pro-B cells (129/Sv). (A–F) Summary of some of the results obtained by SMARD and from the fitting procedure (as described for Figure S2). For three of the restriction fragments analyzed in these cells, the number of double-labeled DNA molecules displayed the occurrence of more than one initiation event is significantly higher than in uncommitted pro-B cells (9%, 14%, and 12%, respectively, for SwaI #2, PmeI #4, and PmeI #5). This is consistent with an increase in the average number of origins firing within the locus during each replication cycle (see main text). (TIF) [file pbio.1001360.s004.tif]

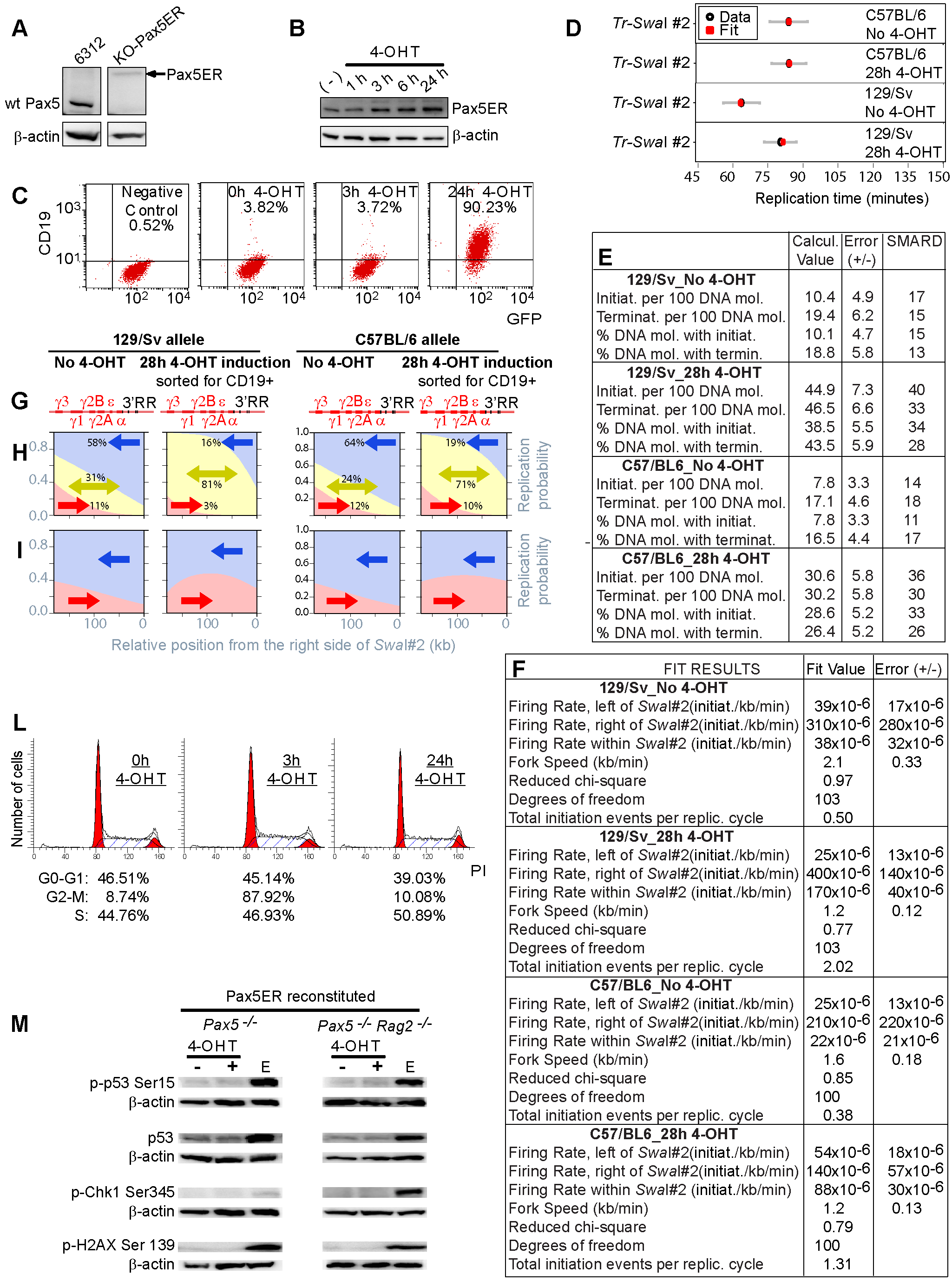

Supplement: Figure S5 — Reconstitution of Pax5 expression in Pax5 −/− uncommitted pro-B cells (129/Sv-C57BL/6). (A) Level of expression of Pax5ER determined using a monoclonal antibody that recognizes the amino-terminal portion of Pax5. Before 4-OHT induction, the level of Pax5ER in KO-Pax5ER pro-B cells is approximately 20% of the level of wt Pax5 present in the pro-B cell line 6312. (B) Following 4-OHT induction, the expression of Pax5ER increases several times, to reach levels comparable to wt Pax5. (C) Kinetics of expression of the surface marker CD19 following 4-OHT induction. A low percentage of CD19+ cells are present before the addition of 4-OHT, but this value increases more than 20 times after the induction of Pax5ER. A small portion of the KO-Pax5ER pro-B cells remains CD19− even after prolonged 4-OHT induction. (D–I) Summary of some of the results obtained by SMARD and from the fitting procedure (as described for Figure S2). (L) Cell cycle profiles of KO-Pax5ER pro-B cells before (0 h) and after 4-OHT induction (3 h and 24 h). These cells continue to cycle even after 4-OHT treatments longer than 1 wk (not shown). (M) Absence of markers of DNA damage and checkpoints'activation following 4-OHT induction. Immunoblots from two different reconstitution experiments (Pax5 −/− and Pax5 −/− Rag2 −/−) are shown before (−) and after (+) a 3 h treatment with 1 μM 4-OHT. Similar results were obtained using induction times between 1 and 24 h, and with antibodies against phospho-ATM Ser1981, and phospho-Rad17 Ser645 (not shown). The positive controls for each immunoblot (E) were prepared by treating KO-Pax5ER pro-B cells with 25 μM etoposide for 2 h. (TIF) [file pbio.1001360.s005.tif]

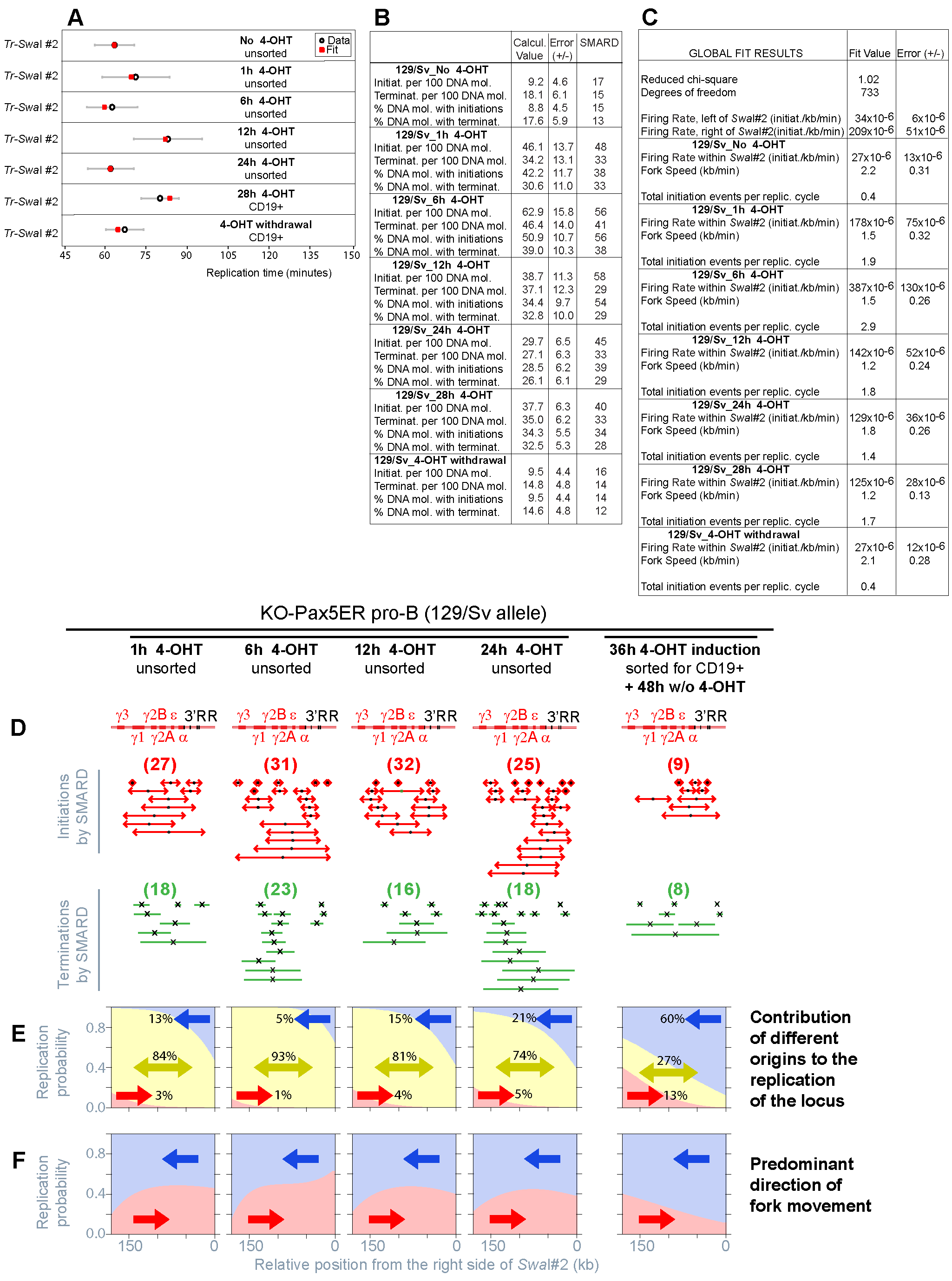

Supplement: Figure S6 — Kinetic of the changes in origin activity induced by 4-OHT (and by 4-OHT withdrawal) within the CH-3′RR region in KO-Pax5ER pro-B cells. (A–F) Summary of some of the results obtained by SMARD and from the fitting procedure (as described for Figure S2). All experimental data sets obtained for the 129/Sv allele were fitted simultaneously as described in Materials and Methods. Panel C also shows the positions of the initiation and termination events detected by SMARD, with numerals indicating the normalized frequency of the events as described for Figure 2. (TIF) [file pbio.1001360.s006.tif]

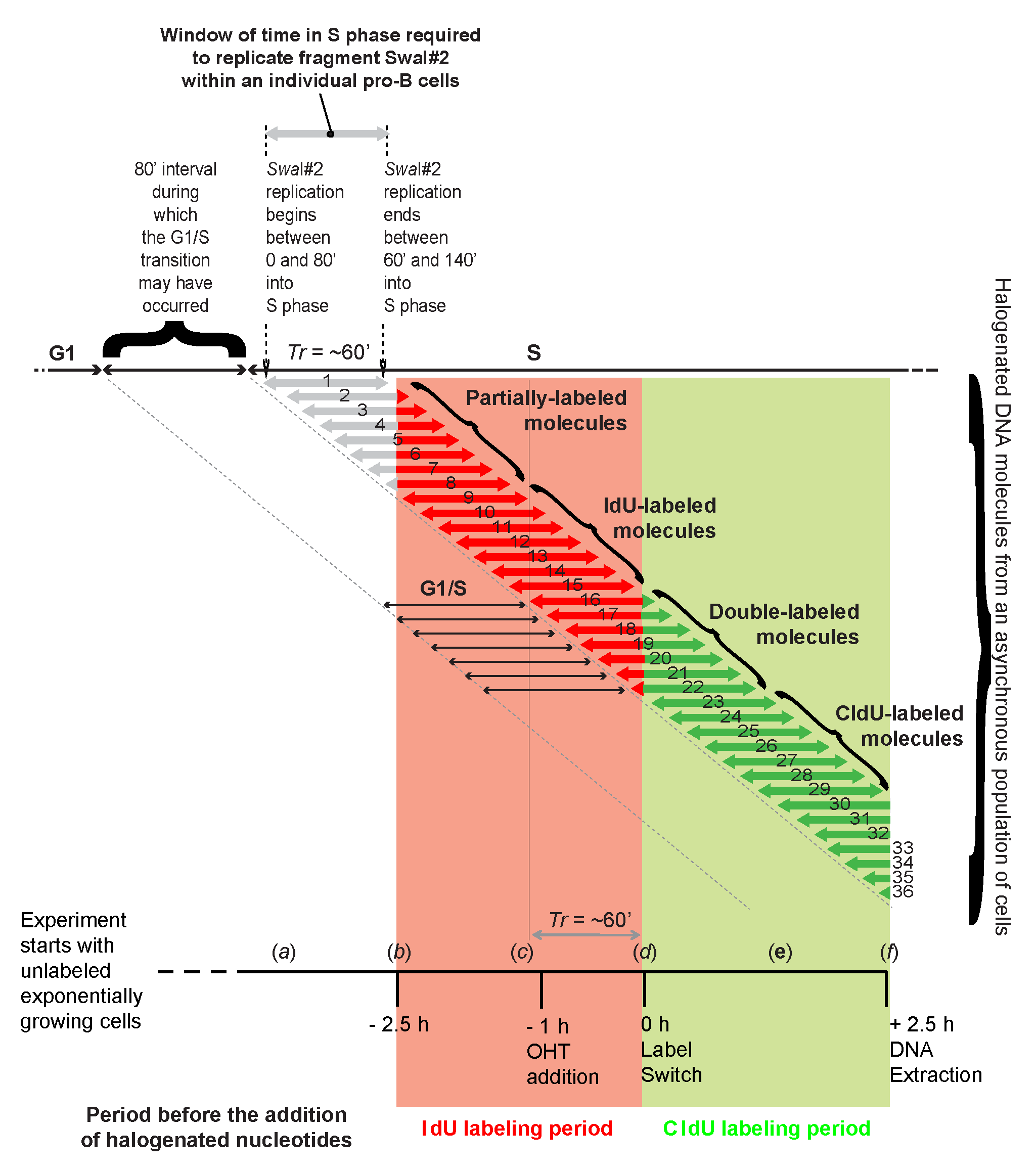

Supplement: Figure S7 — Labeling of DNA molecules from fragment SwaI#2 during SMARD. Results presents in Table 1 indicate that SwaI#2 requires approximately 60′to complete its replication. At the top of the picture, this window of time is presented as a gray double-headed arrow. In an asynchronous population of cells, this window of time will be variably distributed in relation to the labeling periods with IdU and CldU (see double-headed arrows labeled from1 to 36). This will result in molecules that are variably substituted with the nucleotide (unlabelled if the molecule started replicating before time a, partially IdU-labeled if the molecule started replicating between time a and b, fully IdU-labeled between time b and c, double-labeled between time c and d, and so on). For each cell, the window always encompasses a portion of S phase. From Figures, 2F, 4F, and 5H we know that the replication of fragment SwaI#2 is complete in more than 90% of cells by 140′ minutes into S phase. Since the fragment takes approximately 60′ to replicate, we conclude that the replication of each individual molecule starts between 0′ and 80′ (140 – 60 = 80) into S phase (meaning that the G1/S occurs from 0′ to 80′ before each fragment starts replicating). Black double-headed arrows indicate the possible location of the G1/S transition for the cells that produce the population of double labeled molecules. We can see that when 4-OHT is provided 1 h before the label switch a few cells will have already passed the G1/S transition (e.g., #16), while many others will have a similar probability of being at the end of G1 or in early S (e.g., #19–22). (TIF) [file pbio.1001360.s007.tif]
