# Supplementary material for: Regulation of DNA Replication within the Immunoglobulin Heavy-Chain Locus During B Cell Commitment
Source: PLoS Biol. 2012 Jul 10;10(7):e1001360. doi: 10.1371/journal.pbio.1001360 (PMC3393677; doi:10.1371/journal.pbio.1001360)
Supplement: Table S1 — Quantitative analysis of the SMARD data. Column a, genetic background, cell type and treatment, restriction fragment name, and mouse strain(s) of origin for the Igh loci analyzed in each experiment. The name of the mouse strain repeated twice indicates that the cells used for the experiment were homozygous and that the two Igh alleles were analyzed together. In all other cases, the Igh alleles were analyzed independently and only the corresponding mouse strain is indicated. ΔDH-JH indicates that the Igh allele was DH-JH rearranged. Column b, duration of the first and second labeling periods with IdU and CldU (respectively, Tp1 and Tp2). SMARD requires that the duration of each labeling period is longer than the average replication time of the restriction fragments under investigation [17],[22]. Column c, number of Igh molecules surveyed by fluorescence microscopy. This number includes all the molecules detected during each experiment based on the presence of the hybridization signals and regardless of the level of substitution with halogenated nucleotides (e.g., fully, partially, or not substituted). In some of the experiments only the molecules fully substituted with halogenated nucleotides were counted; therefore, this value was not determined (n.d.). Columns d and e, respectively, percentage and number of surveyed molecules containing one or both halogenated nucleotides. Only some of these molecules fulfilled the criteria required by SMARD and were included in column f (see below). Column f, NR, NG, and NRG indicate the number of imaged molecules substituted with, respectively, IdU (detected in red), CldU (detected in green), and both halogenated nucleotides (double-labeled, both red and green). These numbers include only the molecules suitable for measurement (fully substituted, unbroken, sufficiently stretched to be unequivocally aligned to the map of the region, and not overlapping with other molecules). Column g, number of initiation events normalized for 100 [file pbio.1001360.s008.pdf]

SUPPLEMENTARY TABLE 1

(a) Cell Type & Restriction Fragment	*(b)* Labeling time in (minutes) IdU__CldU Tp1___Tp2	*(c)* Total # of *Igh* molecules surveyed	*(d)* % of *Igh* molecules containing halogenated nucleotides	*(e)* Total # of imaged molecules fully substituted with IdU, CldU, or both	*(f)* # of *Igh* molecules suitable for SMARD N_R_N_G_N_{RG}	*(g)* # of initiation events per 100 molecules % of molecules showing initiation events within the fragment	*(h)* # of colliding forks per 100 molecules % of molecules showing colliding forks within the fragment	*(i)* Average time required to replicate the genomic region encompassed by the restriction fragment Tr	*(l)* Average # of replication forks per fragment	*(m)* Average speed per replication fork (kb/min.)	*(n)* Fragment Size (kb)
Pax5$^{-/-}$ **Pro-B cells**											
Pax5$^{-/-}$ ***Rag2***$^{-/-}$ **Bone Marrow** *Swa*I (129/Sv_129/Sv)	225___225	942	33%	311	70__103___36	0<br>0%	39<br>39%	58	1.39	2.25	181
Pax5$^{-/-}$ ***Rag2***$^{-/-}$ **Bone Marrow** *Pac*I (129/Sv_129/Sv)	225___225	1809	33%	597	119__182__57	51<br>51%	0<br>0%	54	1.51	2.24	183
Pax5$^{-/-}$ ***Rag2***$^{-/-}$ **Bone Marrow** *Pme*I-#4 (129/Sv_129/Sv)	225___225	n.d.	n.d.	242	39___30___27	15<br>7%	59<br>48%	107	1.74	2.98	~555
Pax5$^{-/-}$ ***Rag2***$^{-/-}$ **Bone Marrow** *Pme*I-#5 (129/Sv_129/Sv)	225___225	n.d.	n.d.	154	35___28___38	50<br>47%	18<br>18%	130	1.68	2.25	~492
Pax5$^{-/-}$ **(B.M. clone)** *Swa*I (129/Sv, ΔD-J)	240___240	298	39%	116	28___16___23	0<br>0%	0<br>0%	142	1	1.20	181
Pax5$^{-/-}$ **(B.M. clone)** *Pac*I (129/Sv, ΔD-J)	240___240	288	40%	115	41___28___11	0<br>0%	0<br>0%	68	1	1.69	~118
Pax5$^{-/-}$ **(B.M. clone)** *Swa*I (C57BL/6, ΔD-J-r)	240___240	316	37%	117	26___22___19	0<br>0%	11<br>11%	111	1.11	1.31	~168
Pax5$^{-/-}$ **(B.M. clone)** *Pac*I (C57BL/6, ΔD-J-r)	240___240	541	34%	184	44___41___14	14<br>14%	0<br>0%	61	1.14	1.15	~141
wt **Pro-B cells**											
Rag2$^{-/-}$ **Bone Marrow** *Swa*I (129/Sv_129/Sv)	180___180	n.d.	n.d.	227	58___75___34	50<br>41%	24<br>24%	56	1.74	1.86	181
Rag2$^{-/-}$ **Bone Marrow** *Pac*I (129/Sv_129/Sv)	180___180	n.d.	n.d.	302	70__104___66	20<br>20%	20<br>20%	70	1.39	1.88	183
Rag2$^{-/-}$ **Bone Marrow** *Pme*I-#4 (129/Sv_129/Sv)	180___180	n.d.	n.d.	234	25___21___44	59<br>45%	55<br>36%	122	2.14	2.13	~555
Rag2$^{-/-}$ **Bone Marrow** *Pme*I-#5 (129/Sv_129/Sv)	180___180	n.d.	n.d.	210	61___56___69	65<br>55%	55<br>42%	99	2.20	2.26	~492

**Pax5ER Reconstituted Pro-B cells**											
**KO-Pax5ER**<br>**w/o 4-OHT**<br>*Swa*I<br>(C57BL/6)	**220___220**	**774**	**46%**	**356**	**97__115___71**	**14**<br>**11%**	**18**<br>**17%**	**84**	**1.32**	**1.56**	**173**
**KO-Pax5ER**<br>**w/o 4-OHT**<br>*Swa*I<br>(129/Sv)	**220___220**	**808**	**39%**	**315**	**98__113___46**	**17**<br>**15%**	**15**<br>**13%**	**64**	**1.33**	**2.13**	**181**
**KO-Pax5ER**<br>**28h 4-OHT**<br>**CD19+**<br>*Swa*I<br>(C57BL/6)	**220___220**	**n.d.**	**n.d.**	**374**	**102__123__76**	**36**<br>**33%**	**30**<br>**26%**	**84**	**1.64**	**1.26**	**173**
**KO-Pax5ER**<br>**28h 4-OHT**<br>**CD19+**<br>*Swa*I<br>(129/Sv)	**220___220**	**882**	**49%**	**432**	**98__139___80**	**40**<br>**34%**	**33**<br>**28%**	**80**	**1.73**	**1.31**	**181**
**KO-Pax5ER**<br>**28h 4-OHT**<br>**CD19-**<br>*Swa*I<br>(129/Sv)	**220___220**	**n.d.**	**n.d.**	**355**	**84__137___66**	**53**<br>**44%**	**26**<br>**23%**	**72**	**1.79**	**1.40**	**181**
$Pax5^{-/-}$ $Rag2^{-/-}$<br>**Bone Marrow**<br>**36h 4-OHT**<br>**(mock induc.)**<br>*Swa*I<br>(129/Sv_129/Sv)	**180___180**	**363**	**27%**	**98**	**18___24___26**	**0**<br>**0%**	**15**<br>**15%**	**93**	**1.15**	**1.69**	**181**
**KO-Pax5ER**<br>**36h 4-OHT**<br>**CD19+,**<br>**48h w/o 4-OHT**<br>*Swa*I<br>(129/Sv)	**180___180**	**800**	**38%**	**304**	**81___97___58**	**16**<br>**14%**	**14**<br>**12%**	**67**	**1.29**	**2.09**	**181**
**KO-Pax5ER**<br>**w/o 4-OHT**<br>*Swa*I<br>(129/Sv)	**180___180**	**633**	**18%**	**114**	**34___41___16**	**19**<br>**13%**	**25**<br>**25%**	**51**	**1.38**	**2.57**	**181**
**KO-Pax5ER**<br>**1h 4-OHT**<br>*Swa*I<br>(129/Sv)	**180___180**	**787**	**15%**	**118**	**33___32___21**	**48**<br>**38%**	**33**<br>**33%**	**71**	**1.81**	**1.41**	**181**
**KO-Pax5ER**<br>**6h 4-OHT**<br>*Swa*I<br>(129/Sv)	**180___180**	**1131**	**13%**	**147**	**29___60___32**	**56**<br>**56%**	**41**<br>**38%**	**63**	**1.97**	**1.46**	**181**
**KO-Pax5ER**<br>**12h 4-OHT**<br>*Swa*I<br>(129/Sv)	**180___180**	**453**	**17%**	**77**	**20___28___24**	**58**<br>**54%**	**29**<br>**29%**	**83**	**1.88**	**1.16**	**181**
**KO-Pax5ER**<br>**24h 4-OHT**<br>*Swa*I<br>(129/Sv)	**180___180**	**1150**	**22%**	**253**	**64___97___51**	**45**<br>**39%**	**33**<br>**29%**	**62**	**1.78**	**1.64**	**181**
